# Dissecting the Nanoscale Distributions and Functions of Microtubule-End-Binding Proteins EB1 and ch-TOG in Interphase HeLa Cells

**DOI:** 10.1371/journal.pone.0051442

**Published:** 2012-12-12

**Authors:** Satoko Nakamura, Ilya Grigoriev, Taisaku Nogi, Tomoko Hamaji, Lynne Cassimeris, Yuko Mimori-Kiyosue

**Affiliations:** 1 Optical Image Analysis Unit, RIKEN Center for Developmental Biology, Kobe, Hyogo, Japan; 2 Division of Cell Biology, Faculty of Science, Utrecht University, Utrecht, The Netherlands; 3 Department of Biological Sciences, Lehigh University, Bethlehem, Pennsylvania, United States of America; National Cancer Institute, NIH, United States of America

## Abstract

Recently, the EB1 and XMAP215/TOG families of microtubule binding proteins have been demonstrated to bind autonomously to the growing plus ends of microtubules and regulate their behaviour in *in vitro* systems. However, their functional redundancy or difference in cells remains obscure. Here, we compared the nanoscale distributions of EB1 and ch-TOG along microtubules using high-resolution microscopy techniques, and also their roles in microtubule organisation in interphase HeLa cells. The ch-TOG accumulation sites protruded ∼100 nm from the EB1 comets. Overexpression experiments showed that ch-TOG and EB1 did not interfere with each other’s localisation, confirming that they recognise distinct regions at the ends of microtubules. While both EB1 and ch-TOG showed similar effects on microtubule plus end dynamics and additively increased microtubule dynamicity, only EB1 exhibited microtubule-cell cortex attachment activity. These observations indicate that EB1 and ch-TOG regulate microtubule organisation differently via distinct regions in the plus ends of microtubules.

## Introduction

In cells, microtubule dynamics and organisation are controlled by a variety of microtubule regulators. The lengths and positions of microtubules in cells are appropriately controlled by microtubule plus-end-binding proteins that target the microtubule plus ends [1], [2]. Among these molecules, end-binding 1 (EB1) family proteins and XMAP215/TOG family proteins have been demonstrated to autonomously bind to growing microtubule ends and regulate microtubule dynamics in *in vitro* reconstituted systems [3]–[5].

*Xenopus* XMAP215 has been identified as both a stabiliser and destabiliser of microtubules, and is thought to be an important antipause factor that promotes overall microtubule dynamicity [6], [7]. *In vitro* reconstitution studies revealed that XMAP215 binds to microtubule ends and catalyses the addition of tubulin dimers to the growing plus end, while under some circumstances XMAP215 can also catalyse microtubule shrinkage [3], [8]. The mammalian homologue of XMAP215, hepatic tumour overexpressed gene (ch-TOG) [9], also promotes microtubule assembly *in vitro* [10].

EB1 family proteins accumulate at microtubule plus ends in a comet shape exclusively in the growth phase [11], [12]. They are highly conserved members of the microtubule plus end-tracking proteins (+TIPs), which consist of diverse groups of microtubule-binding proteins including cytoplasmic linker protein (CLIP)-170, CLIP-associating proteins (CLASPs), adenomatous polyposis coli (APC) tumour suppressor, and also the microtubule depolymerising kinesin, MCAK (reviewed [1], [13], [14]). In common with XMAP215/TOG family proteins, EB1 family proteins themselves are able to influence microtubule dynamics in cells and *in vitro* systems [5], [15]–[17]. However, EB1 family proteins are distinct in that they act as core components of +TIPs by mediating the tip accumulation of other microtubule modulators with different functions, e.g. microtubule stabilising and destabilising activities. EB1 family proteins can thereby regulate microtubule behaviour differently in different situations [2], [18]. Recently, a well-conserved EB1-recognition mechanism involving a short polypeptide motif, Ser-x-Ile-Pro (SxIP), that enables the accumulation of a variety of proteins with EB1-decorated microtubule ends, has been identified [19], [20] and its biological importance in epithelial morphogenesis confirmed using a three-dimensional culture system [21].

**Figure 1:**
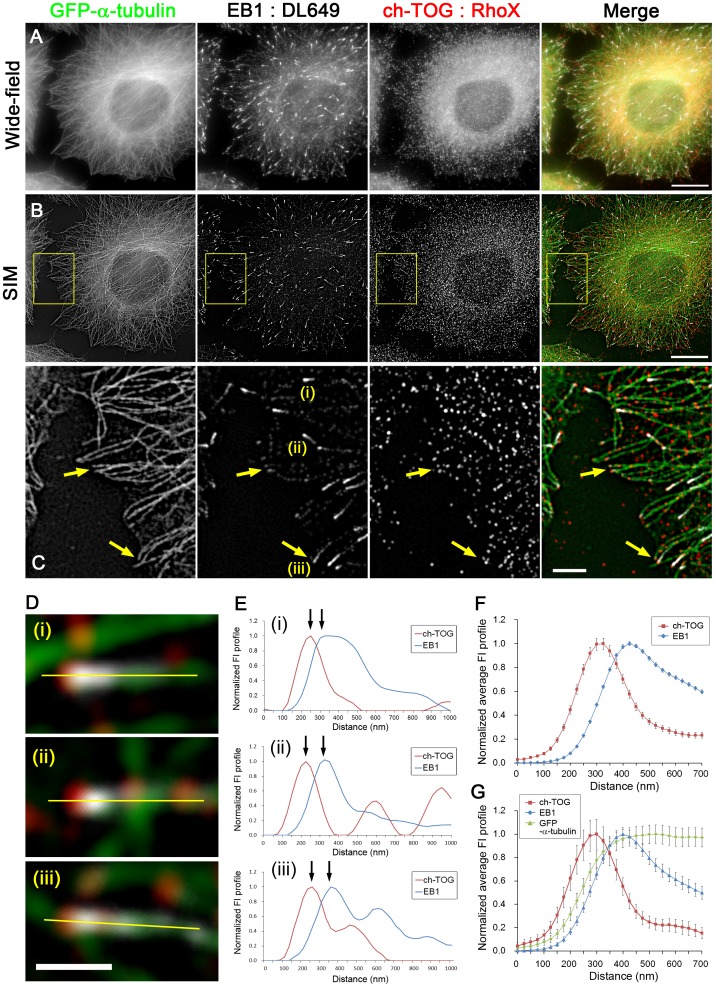
Distributions of EB1 and ch-TOG in HeLa cells. HeLa/GFP-α-tubulin clones (1E10) were immunostained for EB1 and ch-TOG using DyLight 649 (DL649)- and Rhodamine Red-X (RhoX)-conjugated secondary antibodies, respectively, and visualised by wide-field microscopy (**A**) and structured illumination microscopy (SIM) (**B**). The merged images are shown in the right panel, where green indicates GFP-α-tubulin, white indicates EB1 and red indicates ch-TOG. Scale bars, 10 µm. The boxed areas in (B) are enlarged in (**C**). Note that some of the ch-TOG particles are present at more distal sites than EB1. Yellow arrows indicate microtubule ends stained positively for ch-TOG but negatively for EB1. Scale bar, 2 µm. (**D**) Enlarged images of the EB1 comets labelled (i)–(iii) in the EB1 panel in (C). Scale bar, 500 nm. (**E**) Line profiles of fluorescence intensity (FI) plotted from the yellow lines along microtubules indicated in (D). The positions of the peak intensities of EB1 and ch-TOG are separated along the microtubules (arrows). (**F**) Average FI profiles of EB1 and ch-TOG were obtained by analysing multiple SIM images (n = 330, 8 cells in 4 images) and plotted. The data were normalised and the peak intensities were set to 1. The error bars are SEM. (**G**) EB1-positive microtubule ends not overlapping with other microtubules were selected and the FI profiles of EB1, ch-TOG and GFP-α-tubulin were averaged (n = 54, 8 cells in 4 images). The values were normalised and the peak intensities were set to 1. The error bars are SEM.

Despite numerous independent studies describing the actions of EB1 or ch-TOG on microtubule plus end dynamics, their biological functions have not been directly compared. In this study, we compared the microtubule-tip-binding properties and functions of EB1 and ch-TOG in the regulation of microtubule dynamics and organisation in interphase HeLa cells. First, by employing high-resolution structured illumination microscopy (SIM) technique, we showed that ch-TOG binds to more distal sites along the microtubules than EB1 comets in fixed cells. The SIM observations were confirmed in living cells by total internal reflection fluorescence (TIRF) microscopy, which achieves high temporal resolution with high sensitivity. Overexpression studies revealed their binding to non-overlapping regions on the microtubule ends. We next showed that EB1 and ch-TOG have similar effects on overall microtubule dynamicity, while EB1 as well as EB3, but not ch-TOG, exhibited microtubule-cell cortex attachment activity. Our findings provide new insight into the structures of growing microtubule ends and highlight the unique function of EB1 in organising microtubule networks by mediating microtubule plus end-attachment to the cell cortex.

**Figure 2:**
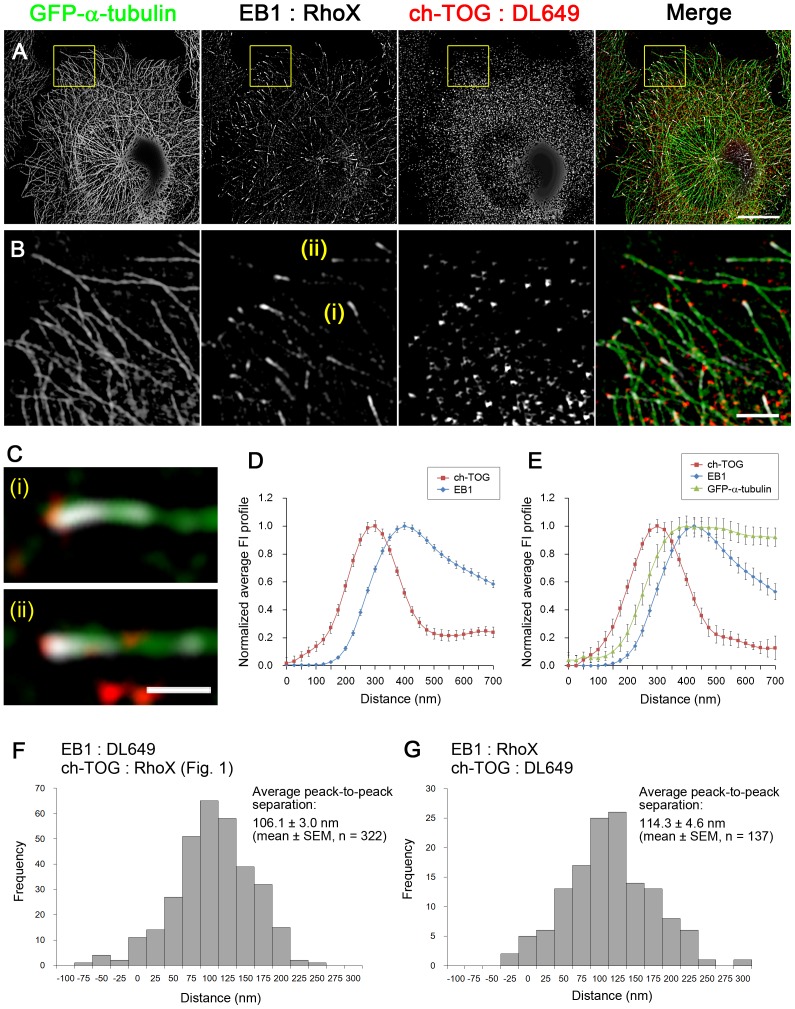
Reciprocal labelling of EB1 and ch-TOG with swapped fluorophores. As a control experiment for Figure 1, the fluorophores of the secondary antibodies used to label EB1 and ch-TOG were swapped. (**A**) The HeLa/GFP-α-tubulin clones (1E10) were immunostained for EB1 and ch-TOG using Rhodamine Red-X (RhoX)- and DyLight 649 (DL649)-conjugated secondary antibodies, respectively, and visualised by SIM. The merged images are shown in the right panel, where white indicates the RhoX channel, red indicates the DL649 channel and green indicates the GFP channel. Scale bars, 10 µm. (**B**) The boxed areas in (A) are enlarged. Scale bar, 2 µm. (**C**) Enlarged images of the EB1 comets labelled (i) and (ii) in the EB1 panel in (C). Scale bar, 500 nm. (**D**) Average FI profiles of EB1 and ch-TOG were obtained by analysing multiple SIM images (n = 139, 4 cells in 3 images) and plotted. The data were normalised and the peak intensities were set to 1. The error bars are SEM. (**E**) EB1-positive microtubule ends not overlapping with other microtubules were selected and the FI profiles of EB1, ch-TOG and GFP-α-tubulin were averaged (n = 44, 4 cells, in 3 images). The values were normalised and the peak intensities were set to 1. The error bars are SEM. (**F**, **G**) Histograms showing the distribution of peak-to-peak distances between EB1 comets and ch-TOG clusters for EB1:DL649 and ch-TOG:RhoX labelling (shown in Figure 1) and for EB1:RhoX and ch-TOG:RhoX labelling (shown in this figure). The EB1 and ch-TOG peak positions were obtained from the line profiles by selecting the ch-TOG peak closest to the EB1 peak. Average peak-to-peak separation was obtained using the values shown in the histogram. There is no statistical significance between the data sets shown in (F) and (G).

## Results

### Comparison of the Nanoscale Distributions of EB1 and ch-TOG in Interphase HeLa Cells

First, we used the high-resolution SIM imaging technique to carefully compare the distributions of endogenous EB1 and ch-TOG at microtubule ends in HeLa cells cultured on collagen-coated cover glasses (Figure S1 and Text S1). This technique can double the spatial resolution of the wide-field epi-fluorescence microscope: it achieves a resolution of ∼100 nm in the lateral direction and ∼300 nm in the axial direction [32], [33]. In addition, we used a technique to measure the separation between protein clusters labelled with multiple different fluorophores at 25-nm resolution in a manner analogous to a method developed to measure average label separation in wide-field images beyond the diffraction-limited resolution [31] (see also Materials and Methods).

**Figure 3:**
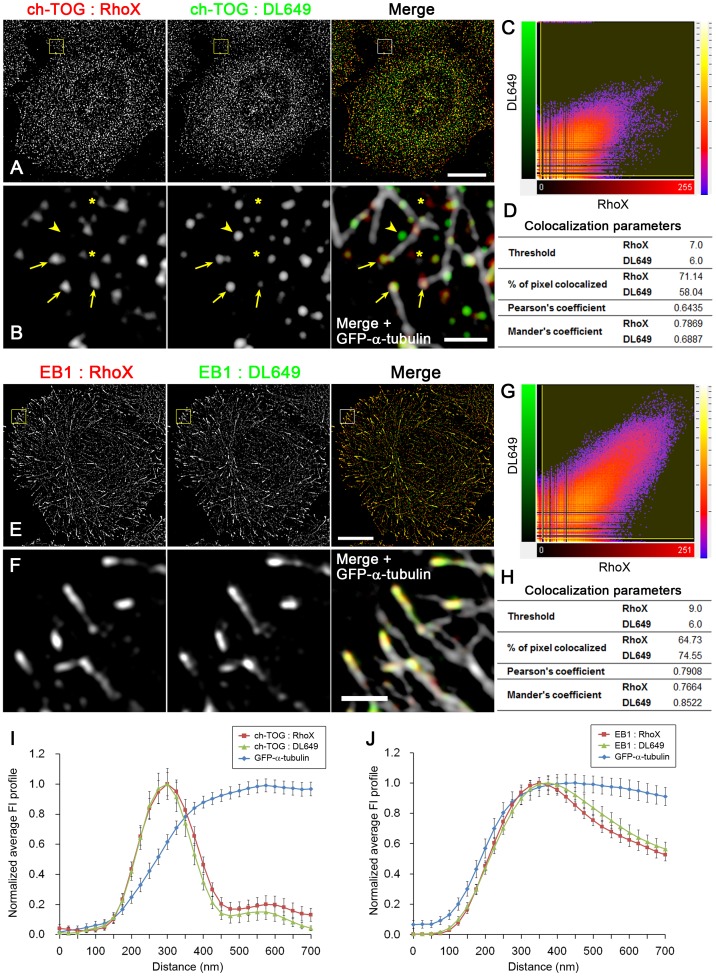
Evaluation of antibodies for EB1 and ch-TOG staining. To test the specificities of the antibodies and the effects of fluorophores (emission wavelength), ch-TOG (**A**) and EB1 (**E**) were labelled with two different secondary antibodies conjugated with Rhodamine Red-X (RhoX) or DyLight 649 (DL649), and visualised by SIM. The RhoX and DL649 channels are merged in the right panels in red and green, respectively. In (**B**) and (**F**), boxed areas in (A) and (E), respectively, are enlarged and merged in the right panels together with GFP-α-tubulin signals (white). In (B), both RhoX and DL649 signals are detected in the same clusters localised at microtubule ends (arrows), although their signal intensities are not necessarily equal probably owing to competing binding to the same antigen, while clusters positive only for RhoX (asterisks) or DL649 (arrowheads) are also observed. The colocalisation of RhoX and DL649 signals was analysed and the scatter plots of the pixel intensities for the RhoX and DL649 channels and their colocalisation parameters are shown, respectively, in (**C**) and (**D**) for ch-TOG and in (**G**) and (**H**) for EB1. The values indicating the colocalisation of the RhoX and DL649 channels show that 30–40% of pixels were not colocalised when analysed at the individual pixel level. However, considering that the same cluster often appears to be a different size when labelled with two different antibodies as shown in (B), the colocalisation percentage may be underestimated. (**I**) Using the ch-TOG-double labelling images including (A), ch-TOG-positive microtubule ends not overlapping with other microtubules were selected and the FI profiles were averaged (n = 52, 5 cells in 3 images). The error bars are SEM. (**J**) Using the EB1-double labelling images including (E), EB1-positive microtubule ends not overlapping with other microtubules were selected and the FI intensities were averaged (n = 52, 2 cells in 2 images). The error bars are SEM.

**Figure 4:**
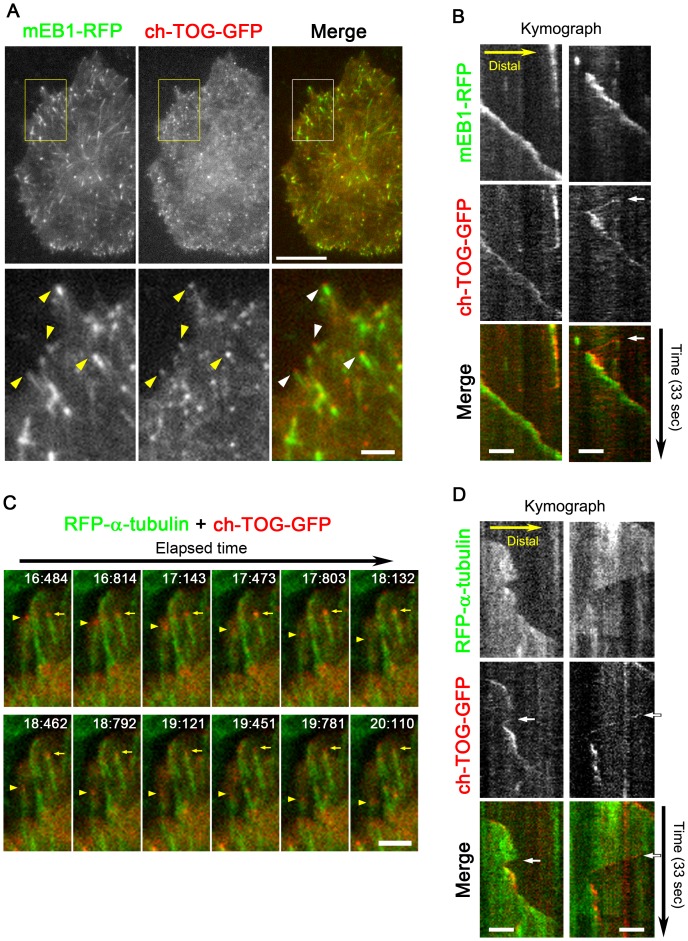
Behaviour of EB1 and ch-TOG in living HeLa cells observed by TIRF microscopy. (**A**) The distributions of mEB1-RFP and ch-TOG-GFP in a living HeLa cell. The merged images are shown in the right panel, where green indicates mEB1-RFP and red indicates ch-TOG-GFP. Scale bar, 10 µm. The boxed areas in the upper panels are enlarged in the bottom panels. See also Movie S1, S2. Scale bar, 2 µm. (**B**) Kymographs showing the movements of ch-TOG-GFP and mEB1-RFP. The kymographs were generated from time-lapse sequences (33-sec long). Two example images are shown. The GFP (red) and RFP (green) signals are merged in the bottom panels. Scale bars, 2 µm. (**C**) Time-lapse series of part of a movie (Movie S3, S4) showing living HeLa cells expressing RFP-α-tubulin (green) and ch-TOG-GFP (red). Growing and shrinking microtubule ends, with bound ch-TOG-GFP, are indicated by the yellow arrows or arrowheads, respectively. The elapsed time is indicated in the figure as s:ms. Scale bar, 2 µm. (**D**) Kymographs showing the movements of ch-TOG-GFP and RFP-α-tubulin are presented similarly to in (B). Scale bar, 2 µm. In all figures, GFP and RFP are pseudo-coloured in red and green, respectively, for consistency with the other images. In the kymographs, the ch-TOG-GFP signals were always detected at a position slightly distal to the EB1 signals (B), although fast retrograde movement of ch-TOG-GFP was also observed (arrows in B). Simultaneous visualisation of ch-TOG-GFP and RFP-α-tubulin demonstrated the tracking of shortening microtubule ends by ch-TOG-GFP (arrows in D).

Co-immunostaining of endogenous EB1 and ch-TOG is shown in Figure 1A, in which microtubules are visualised by expression of exogenous GFP-α-tubulin (Figure S2A, S3, S6B; Text S1). The SIM images of the same field of view are shown in Figure 1B. Although the ch-TOG signals were detectable throughout the cytoplasm, ch-TOG spots located in the vicinity of the tips of EB1 comets were also identified by careful inspection (Figure 1C, D). Interestingly, the position at which the peak intensity of ch-TOG staining was observed was more distal along the microtubules than that of EB1 (Figure 1D, E). The averaged fluorescence intensity profiles of EB1 and ch-TOG are shown in Figure 1F. In Figure 1G, the average profile of the GFP-α-tubulin signal is also shown. Analysis of numerous EB1 comets in multiple SIM images indicated a peak-to-peak separation between EB1 and ch-TOG of 106.1 ± 3.0 nm (mean ± SEM, n = 322, 8 cells in 4 images). The ch-TOG accumulation sites extended up to ∼200 nm from the EB1 comets (Figure 2F).

**Figure 5:**
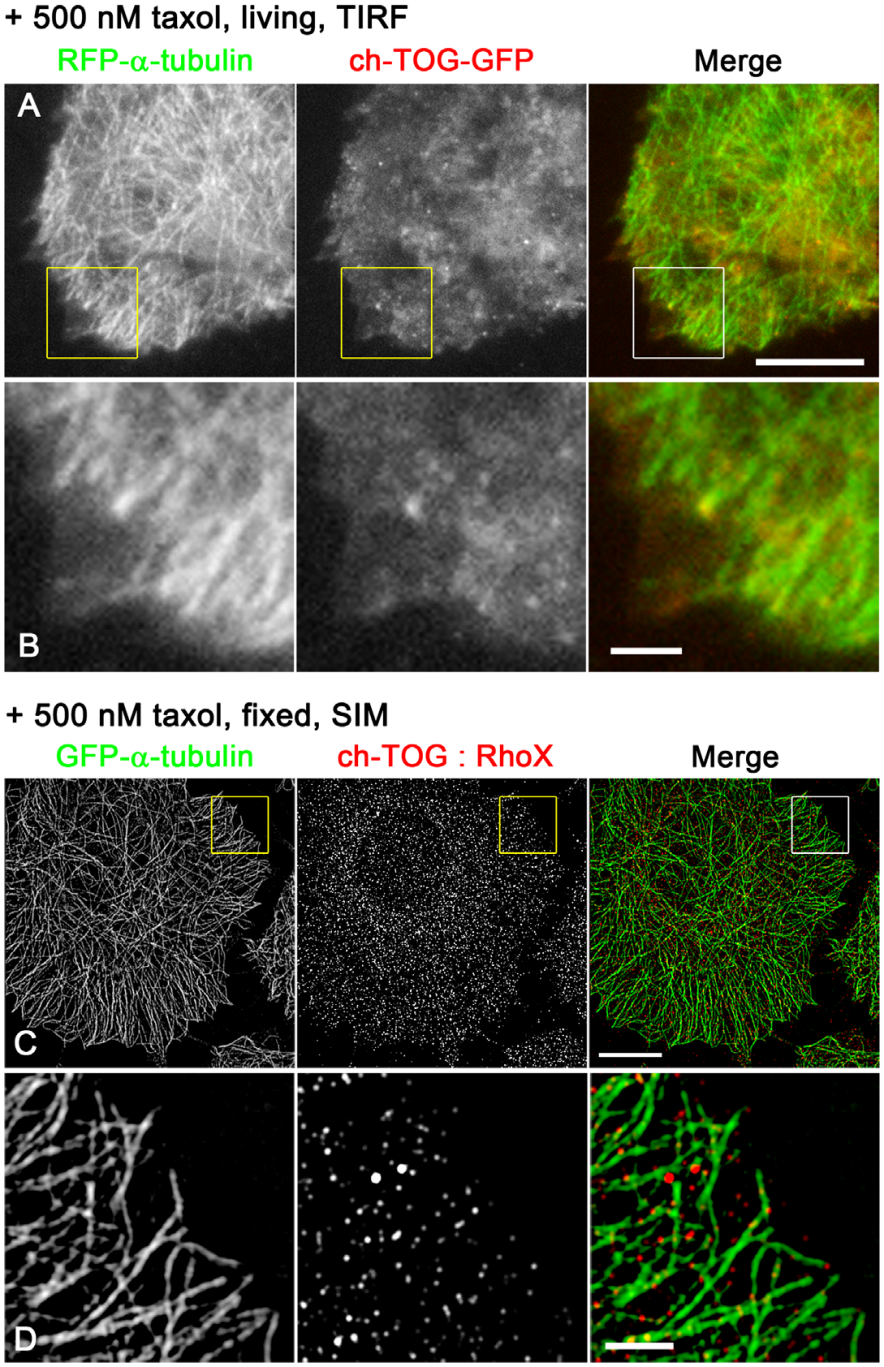
Effect of inhibiting microtubule dynamics on ch-TOG localisation. (**A, B**) ch-TOG-GFP (red) was expressed in HeLa cells expressing RFP-α-tubulin (green) and a low concentration of taxol (500 nM) was added. The living cells were observed by TIRF. The boxed area in (A) is enlarged in (B). Scale bars; 10 µm (A), 2 µm (B). (**C, D**) A HeLa cell clone expressing GFP-α-tubulin (1E10, green) was fixed and stained for endogenous ch-TOG using a secondary antibody conjugated with RhoX (red), and observed by SIM. The boxed area in (C) is enlarged in (D). Scale bars; 10 µm (A), 2 µm (B).

To test if the signal distributions observed were artefacts arising from the optical systems, we repeated the experiments with secondary antibodies conjugated to a different fluorophore (Figure 2). The different fluorophore gave the same results: the average peak-to-peak separation between EB1 and ch-TOG was 114.3 ± 4.6 nm (mean ± SEM., n = 137, 4 cells in 3 images). In addition, we used the two fluorophores to label the same protein to check the specificity of the antibodies and confirm that the two signals were precisely colocalised at microtubule ends, although each channel contained ∼30% non-specific background noise (Figure 3). Therefore, even with ∼30% random background noise, the antibodies precisely visualised EB1 and ch-TOG at the microtubule ends and thus the averaging procedure is expected to reduce noise. Indeed, we reproducibly obtained quite similar average fluorescence intensity profiles under different conditions (Figure 1F, 2D, 3I, 3J). We concluded that our analysis provided a precise spatial relationship between EB1 and ch-TOG in fixed cells.

**Figure 6:**
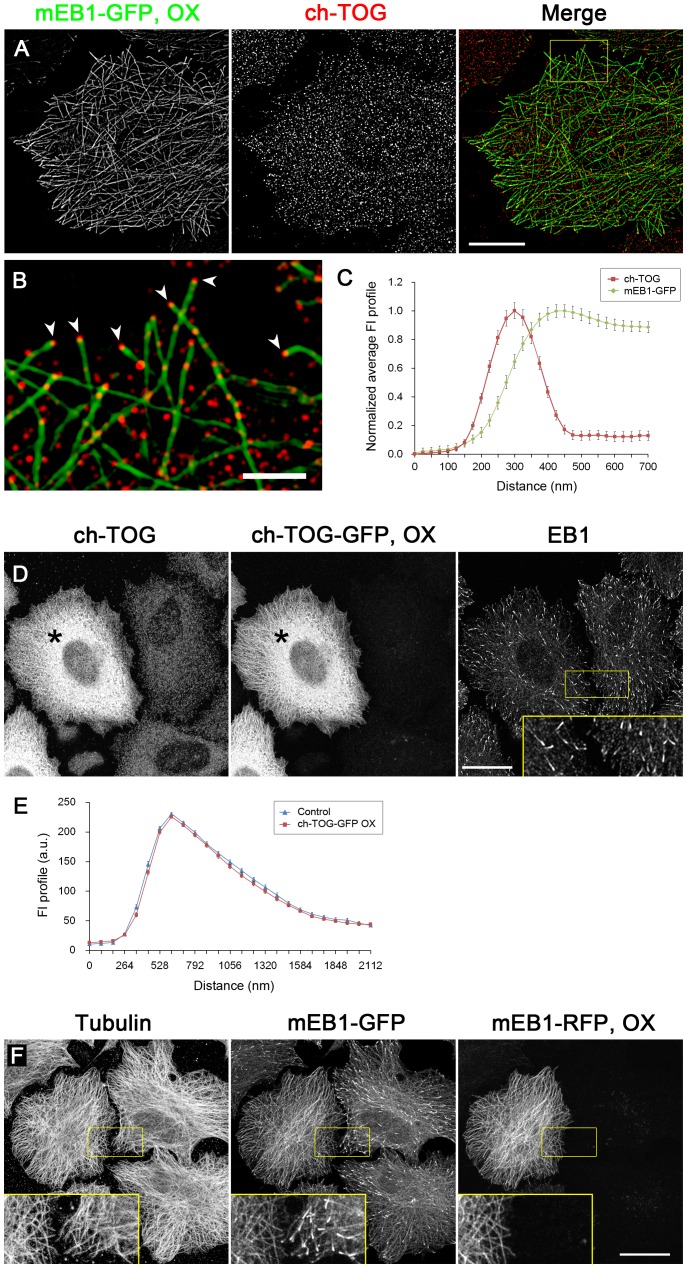
EB1 and ch-TOG do not affect the binding of each other to microtubules. The effects of overexpression of mEB1-GFP or ch-TOG-GFP on the distributions of endogenous ch-TOG or EB1, respectively, were examined. (**A**, **B**) mEB1-GFP (green) was transiently expressed in HeLa cells, and the cells were fixed and stained for endogenous ch-TOG using a RhoX-conjugated secondary antibody (red). To analyse the effect of mEB1-GFP overexpression on the distribution of ch-TOG, cells expressing a large amount of mEB1-GFP that distributed throughout the entire microtubule lattice, in which microtubule ends were expected to be saturated with EB1, were selected and the images were acquired on a SIM microscope. The boxed areas in (A) are enlarged in (B). Microtubule ends positive for ch-TOG clusters are indicated by arrowheads. Scale bars, 10 µm (A), 2 µm (B). (**C**) Average FI profiles of overexpressed mEB1-GFP and ch-TOG were obtained by analysing multiple SIM images (n = 71, 4 cells in 4 images) and plotted. The data were normalised and the peak intensities were set to 1. The error bars are SEM. (**D**) ch-TOG-GFP was exogenously expressed in HeLa cells, and the cells were fixed and stained for ch-TOG and EB1. The images were acquired on a confocal microscope. The expression level of exogenous ch-TOG-GFP was determined by comparing the fluorescence intensity of ch-TOG staining in GFP-negative and positive cells. The cell indicated by the asterisks has an ∼8-fold higher expression level of ch-TOG than untransfected cells. The inset shows the boxed areas at 2.5× magnification. Scale bar: 20 µm. In (**E**), average FI profiles of EB1 in untransfected control cells and ch-TOG-GFP expressing cells (> 6-fold overexpression) were obtained by analysing multiple confocal images (n = 132, 7 cells in 3 images for control; n = 135, 5 cells in 3 images for ch-TOG-GFP expressing cells) and plotted without normalization. The comet-like distribution of EB1, as well as the fluorescence intensity of the EB1 comet, was not altered by ch-TOG-GFP overexpression. (**F**) A control experiment showing competitive binding of EB1 to microtubule ends. mEB1-RFP was transiently overexpressed at a level at which it distributes throughout entire microtubule lattices in HeLa/mEB1-GFP clones (2F10) stably expressing mEB1-GFP at ∼40% the level of endogenous EB1 (Figure S2D, S6E). In RFP-negative cells, mEB1-GFP distributes in a typical comet shape. Overexpression of mEB1-RFP inhibited the accumulation of mEB1-GFP to microtubule ends and redistributed it throughout the entire microtubule lattice. The insets show the boxed areas at 2.5× magnification. Scale bar: 20 µm.

In the SIM images analysed, ch-TOG signals were detectable at the tips of more than 90% of EB1 comets, although the percentage varied to some extent among cells. Furthermore, ch-TOG spots were also found at EB1-negative microtubule ends (Figure 1C, arrows), presumably in microtubules undergoing shrinkage. Live imaging of ch-TOG-GFP behaviour (Figure S4; Text S1) by TIRF microscopy to achieve high temporal resolution with high sensitivity confirmed the spatial relationship between EB1 and ch-TOG. During microtubule growth, the ch-TOG-GFP spots preceded the EB1 comets (Figure 4A, B; Movie S1, S2). Simultaneous visualisation of ch-TOG-GFP and RFP-α-tubulin showed that the ch-TOG-GFP spots track both growing and shrinking microtubule ends (Figure 4C, D; Movie S3, S4), similar to previous observations from an *in vitro* system (XMAP215) and in *Drosophila* cells (*Drosophila* XMAP215 homologue Mini spindles (Msps)) [3], [34]. To further confirm the binding of ch-TOG to microtubule ends, microtubule dynamics were inhibited by addition of a low concentration of taxol (500 nM), a concentration that stops growth/shortening dynamics without inducing substantial alteration of the overall microtubule network pattern, and the cells were observed by TIRF and SIM (Figure 5). In living cells visualised by TIRF, ch-TOG-GFP was absent from the majority of microtubule ends, while ch-TOG-GFP clusters were detectable near a subset of microtubule ends (Figure 5A, B). Consistently, in SIM images of fixed and immunostained cells, ch-TOG signals were undetectable at the majority of microtubule ends (Figure 5C, D). Taken together, we concluded that the immunostaining images precisely represented the relative positions of EB1 and ch-TOG.

**Figure 7:**
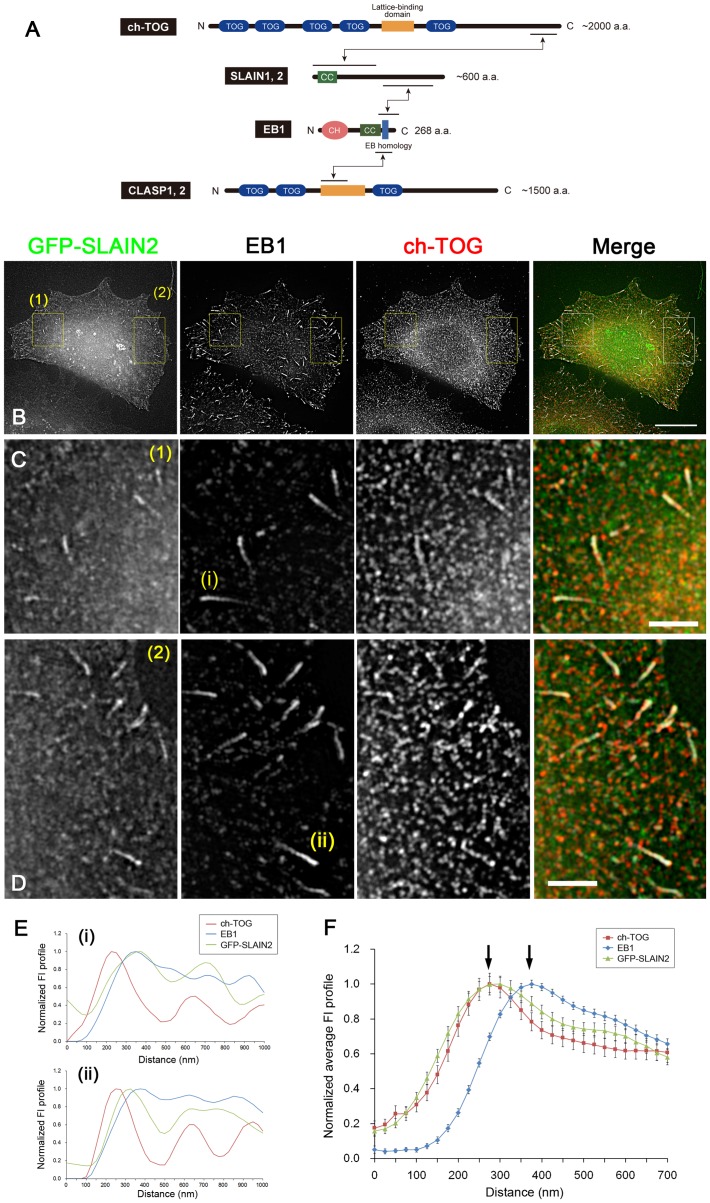
Overexpression of SLAIN2 enhanced the colocalisation of ch-TOG with EB1 comets. (**A**) Schema showing the domain structures of ch-TOG, SLAIN proteins, EB1 and CLASP proteins. Mutual binding sites are indicated by the arrows [23], [24]. (**B**) GFP-SLAIN2 (green) was transiently expressed in HeLa cells, and the cells were fixed and stained for endogenous EB1 (white) and ch-TOG (red). The images were acquired on a SIM microscope. The signals are merged in the right panel. Scale bar, 10 µm. In (**C**) and (**D**), magnified images of the boxed areas (1) and (2) in (B), respectively, are shown. Scale bars, 2 µm. (**E**) The line profiles along the EB1 comets marked with (i) and (ii) in (B) and (C) are plotted. Note that the fluorescence intensity of ch-TOG at the rear part of microtubules does not fall to zero, unlike in SLAIN2-untransfected cells (Figure 1E). (**F**) Average FI profiles of EB1 and ch-TOG were obtained by analysing multiple SIM images (n = 79, 2 cells in 2 images) and plotted. The data were normalised and the peak intensities were set to 1. The error bars are SEM. Note that the ch-TOG distribution extends towards the rear of the microtubules and is co-localised with EB1 comets, while the strong ch-TOG peak is still present at the tips in front of the EB1 comets (arrows). GFP-SLAIN2 distributes on both ch-TOG clusters at the tips and EB1 comets. The difference in the ch-TOG distributions on the EB1 comets in the data sets shown in (F) and in Fig. 1F (as a control) was statistically significant (P < 0.01 at 25 nm rear of the ch-TOG peak, P < 0.001 at > 50 nm rear of the ch-TOG peak).

**Figure 8:**
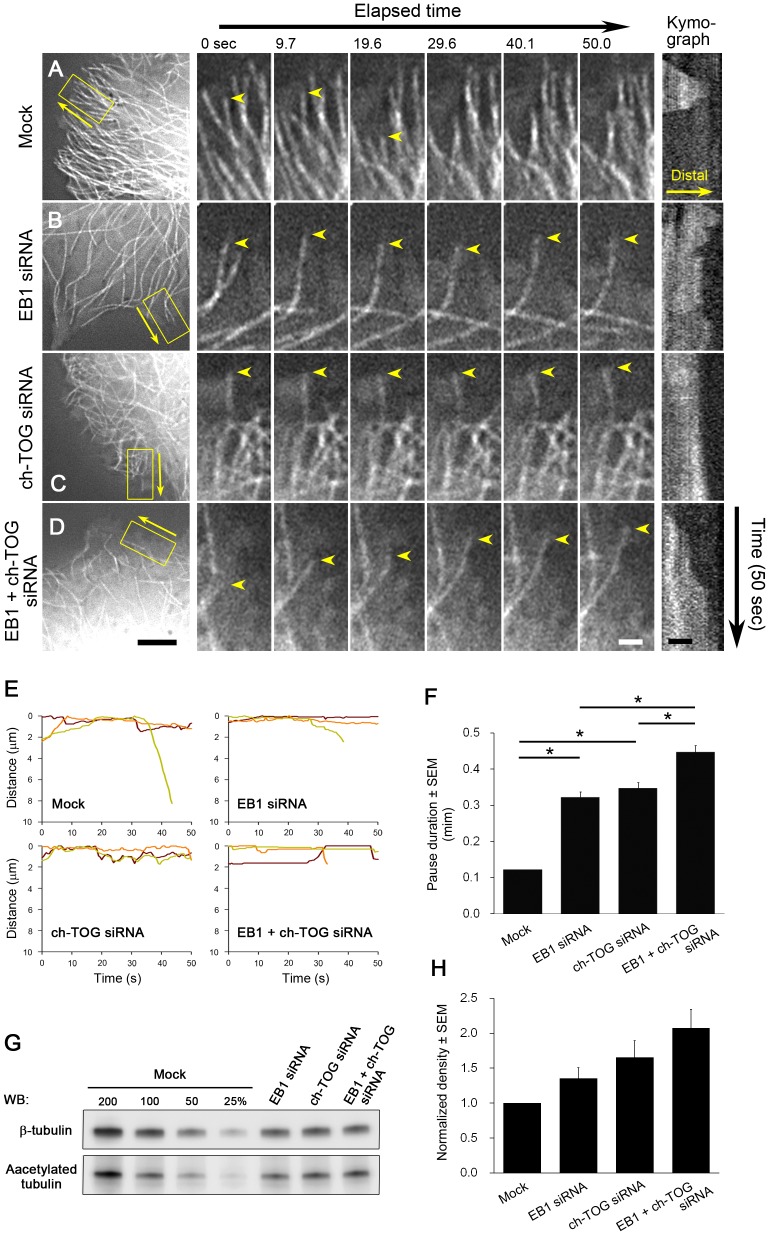
Impact of EB1 and/or ch-TOG depletion on microtubule dynamics in interphase HeLa cells. (**A–F**) Analysis of microtubule dynamics by time-lapse imaging of HeLa/GFP-α-tubulin clones (1E10) after transfection with the indicated siRNAs. Because ch-TOG depletion impairs mitosis and induces dell death, siRNA-treated cells were blocked in interphase 24 h after transfection by addition of thymidine (2.5 mM). Cells were imaged with a 0.5 s interval (Movie S5). In A–D, the first frames of the time-lapse movies (left), selected time-lapse images of the boxed areas (middle), and kymographs of the indicated microtubules (arrowheads) are shown (right). The time-lapse images are arranged with the microtubule distal ends upwards (the direction of the arrows in the left panels). Scale bars; 5 µm (left), 1 µm (middle and right). (**E**) Life-history plots of individual microtubules are shown. Plots for three different MTs are shown by the red, green and orange lines. In (**F**), the duration time of the “pause” state, in which microtubules exhibit no detectable growth/shortening or repeated growth/shortening within a limited distance (< 1 µm), was measured and plotted. The results are presented as means ± SEM (*, P < 0.01; n = 360 in 15 cells for mock, n = 204 in 15 cells for EB1 siRNA, n = 204 in 15 cells for ch-TOG siRNA, n = 179 in 15 cells for EB1 + ch-TOG siRNA). The pause duration time was increased additively following EB1 and ch-TOG double knockdown. (**G**, **H**) Western blotting (WB) analysis for acetylated tubulin, a marker of stable microtubules. In (G), a representative western blot of lysates from cells treated with the indicated siRNAs probed with the indicated antibodies is shown. The average densities of the acetylated tubulin bands obtained from five independent experiments are plotted in (H). The data were normalised and the density of the mock control was set as 1. The results are presented as means ± SEM (P = 0.061 for mock v.s. EB1 siRNA, P = 0.034 for mock v.s. ch-TOG siRNA, P = 0.006 for mock v.s. EB1+ch-TOG siRNA, P = 0.317 for EB1 siRNA v.s. ch-TOG siRNA, P = 0.042 for EB1 siRNA v.s. EB1+ch-TOG siRNA, P = 264 for ch-TOG siRNA v.s. EB1+ch-TOG siRNA; n = 7). For WB full scans, see also Figure S6A.

**Figure 9:**
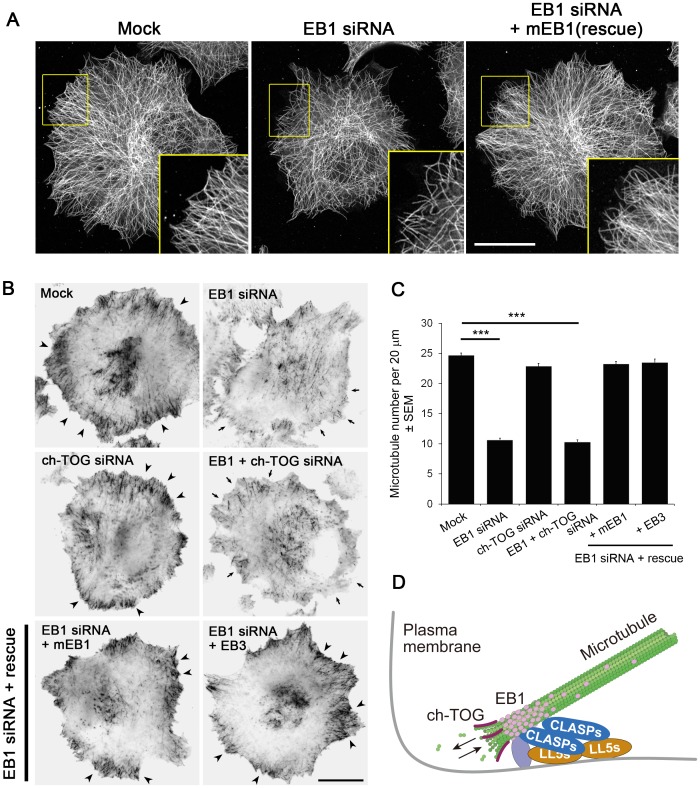
Impact of EB1 and/or ch-TOG depletion on microtubule organisation in interphase HeLa cells. (**A**) Parental HeLa cells and mEB1-expressing HeLa cells were treated with EB1 siRNA, fixed and stained for tubulin, and observed using a confocal microscope. Note that the microtubule density was reduced at the cell periphery in the EB1-depleted cells, while the expression of mEB1 rescued the phenotype. Scale bar, 20 µm. (**B**) The HeLa/RFP-α-tubulin clone (1A2) was treated with the indicated siRNAs and observed by TIRF microscopy. Many microtubules are visible at the cell periphery in cells expressing EB1 (arrowheads), but not in EB1-depleted cells (arrows). For rescue experiments, mEB1 or EB3 were stably introduced into the HeLa/RFP-α-tubulin clone (1A2) and treated with siRNAs against endogenous EB1. In both mEB1- and EB3-expressing cells, the microtubule density at the cell periphery was restored. Scale bar, 20 µm. (**C**) The microtubule numbers visualised by TIRF were analysed and plotted. Values significantly different from control are indicated with asterisks (***, P < 0.001; n = 146 in 41 cells for mock, n = 143 in 33 cells for EB1 siRNA, n = 106 in 39 cells for, n = 126 in 33 cells for EB1 + ch-TOG siRNA, n = 112 in 32 cells for EB1 siRNA + mEB1, n = 102 in 32 cells for EB1 siRNA + EB3). (**D**) A schema depicting EB1 function in the microtubule-anchoring process. EB1 binds to cortical +TIPs, including CLASPs, to mediate microtubule tethering. The linkage between EB1 and the cortex should still allow for ch-TOG-mediated tubulin addition at the very ends of the microtubules.

### Tubulin Distributes in Front of EB1

In all of the averaged fluorescence intensity profiles representing the distribution of EB1 and GFP-α-tubulin signals (Figure 1G, 2E, 3J), GFP signals were detected in front of EB1 signals. The GFP-α-tubulin signals gradually decay from the peak position of EB1 towards the tip, suggesting that the microtubule ends are tapered and ch-TOG binds to the most extended end. The relative distribution of ch-TOG and GFP-α-tubulin is considered to be shown most precisely in Fig. 3I, in which the intensity profiles were aligned at the ch-TOG peak during the averaging process. Because it is possible that the distance between ch-TOG and EB1 localization sites varies at each microtubule end, and therefore in other plots in which the intensity profiles were aligned at the EB1 peak, the shapes of ch-TOG and GFP-α-tubulin may be blurry after applying the averaging procedure. Indeed the ch-TOG peak is the sharpest in the plot shown in Fig. 3I. In this plot, the GFP-α-tubulin signals gradually decay and disappear at the same point as ch-TOG on the distal side.

**Figure 10:**
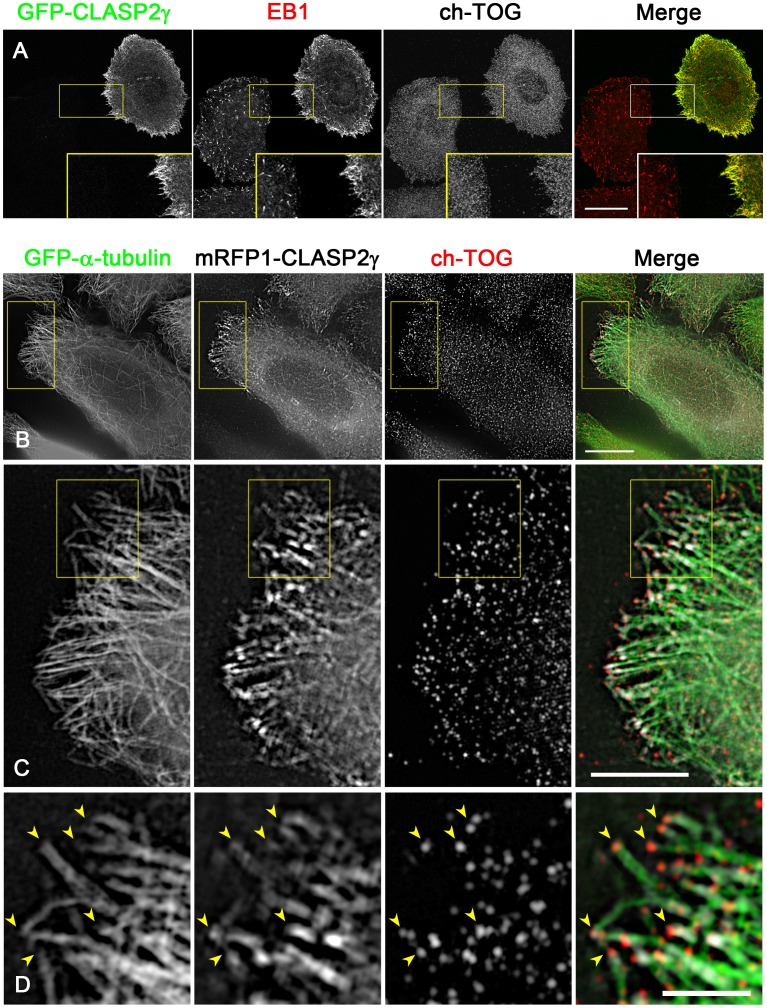
EB1, but not ch-TOG, is recruited to CLASP-accumulating microtubule ends. (**A**) GFP-CLASP2γ was expressed in HeLa cells, and the cells were fixed and stained for endogenous EB1 and ch-TOG. The images were acquired by confocal microscopy. The GFP-CLASP2γ (green) and EB1 (red) signals are merged in the right panel. The insets show the boxed areas at 2× magnification. Note that only EB1, and not ch-TOG, was recruited to the GFP-CLASP2γ-accumulating microtubule ends. Scale bar, 20 µm. (**B–D**) mRFP1-CLASP2γ (white) was expressed in HeLa cell clones (1E10) expressing GFP-α-tubulin (green), and the cells were fixed and stained for endogenous ch-TOG (red). The images are merged in the right panel. The images were acquired by SIM. In the bottom panels, the boxed areas in the upper panels are magnified. Boxed areas in (B) and (C) are enlarged in (C) and (D), respectively. Note that ch-TOG distributes at the most distal portion of microtubule ends even after mRFP1-CLASP2γ overexpression. Scale bars, 10 µm (B, upper), 2 µm (B, bottom), 500 nm (C).

### EB1 and ch-TOG Bind Independently to Microtubule Ends

Next, to examine whether EB1 and ch-TOG compete for binding to microtubules, overexpression experiments were performed. Following expression of EB1-GFP or ch-TOG-GFP in HeLa cells, the cells were fixed and stained for endogenous ch-TOG or EB1, respectively. As shown in Figure 6A–C, when EB1-GFP was overexpressed at a level at which it saturated the microtubule ends and distributed along the entire length of the lattices, the ch-TOG spots remained attached to the microtubule tips. In turn, the EB1 distribution was not affected by overexpression of ch-TOG-GFP (Figure 6D, E). As a control, Figure 3F shows that mEB1-GFP redistributed to the lattice when displaced from microtubule ends by overexpression of mEB1-RFP. The distributions of endogenous EB1 and ch-TOG to microtubule ends were not inhibited by siRNA-mediated depletion of ch-TOG or EB1, respectively (data not shown). Therefore, EB1 and ch-TOG can bind independently to microtubule ends by recognising different regions of microtubule ends.

**Figure 11:**
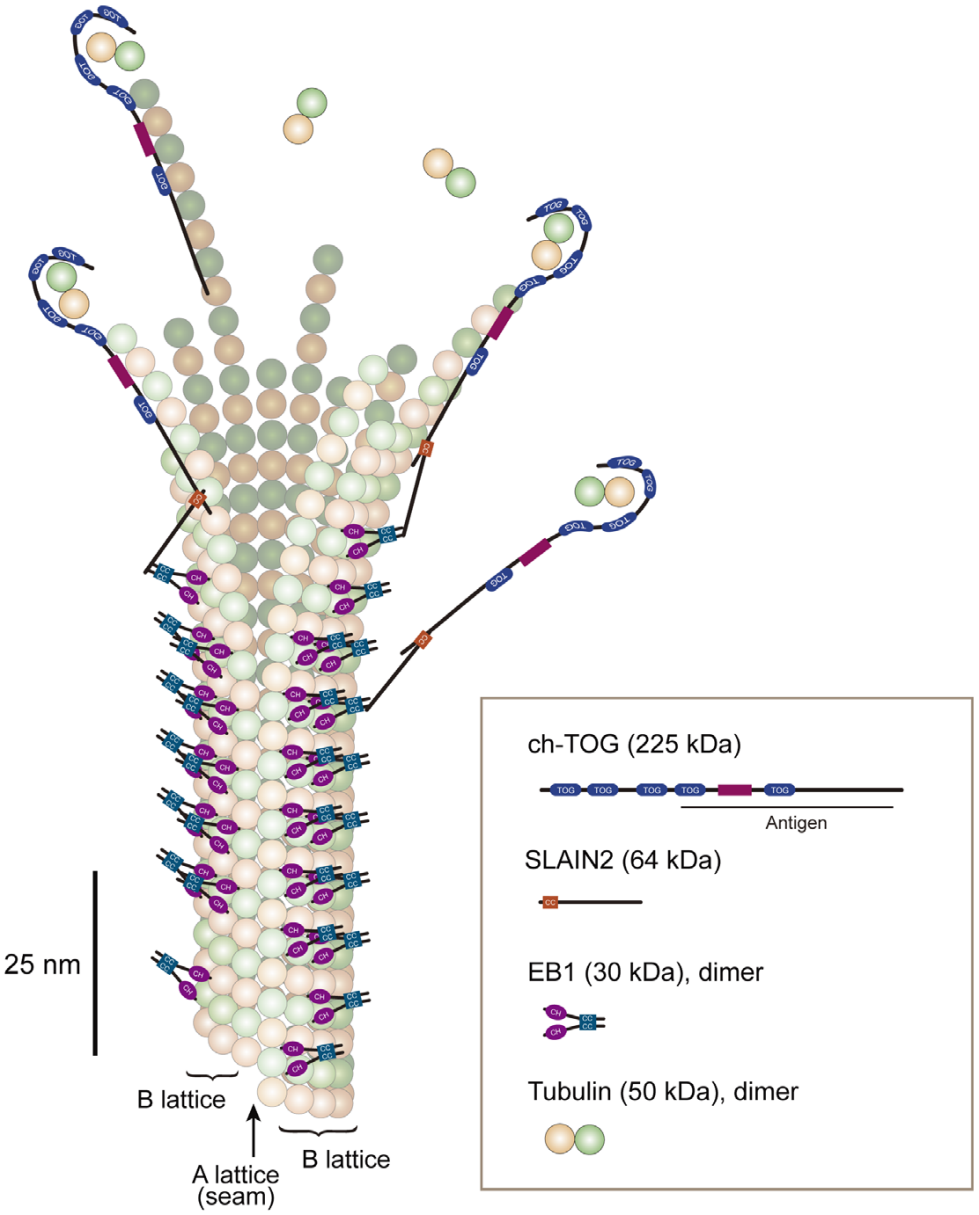
Model of the binding sites for EB1 and ch-TOG. EB1 and ch-TOG are displayed on a graphic of a growing microtubule structure. The shape of the tubulin/ch-TOG complex was adapted from ref. [3]. XMAP215, which has a similar domain structure to ch-TOG, is a long molecule of ∼60 nm [55] that binds small tubulin oligomers [46] or one free tubulin dimer [3] at its NH_2_-terminus. The antibody against ch-TOG was generated using the COOH-terminal half of this molecule [28]. EB1 binds to the closed B lattice of the microtubule wall [56]. The microtubule tip probably contains protofilaments of different lengths. Based on our observation that the peak intensity of ch-TOG and EB1 comets was separated by ∼100 nm, the longer protofilaments for which growth is accelerated by ch-TOG [47] are assumed to consist of ∼12 tubulin dimers. SLAIN proteins may recruit ch-TOG to EB1 comets to increase the local concentration of ch-TOG.

### SLAIN2 Protein Recruits ch-TOG to the EB1 Comets without Affecting its Tip Localisation

A recent report demonstrated that SLAIN proteins link EB1 and ch-TOG (Figure 7A), and these ternary protein complexes distribute along microtubule ends in a typical comet shape and strongly stimulate processive microtubule polymerization [24]. Also, in *Drosophila* S2 cells, Sentin protein mediates the accumulation of Msps, an XMAP215 homologue, to the EB1 comets [35]. In contrast, the comet-like distribution of ch-TOG was not obvious in our observations of HeLa cells. However, when GFP-SLAIN2 was exogenously expressed, endogenous ch-TOG was recruited to EB1 comets together with GFP-SLAIN2, while the ch-TOG signal was still detectable in the more distal regions of EB1 comets (Figure 7B–F) with a peak-to-peak separation between EB1 and ch-TOG of 98.36 ± 6.44 nm (mean ± SEM, n = 76, 2 cells in 2 images), which was not significantly different to the values obtained in cells without exogenous GFP-SLAIN2. It is assumed that the primary binding site for ch-TOG is the tips of microtubules, and that SLAIN proteins retain ch-TOG on the EB1 comet probably to increase local concentration of ch-TOG. These results suggest that the endogenous SLAIN activity or abundance in HeLa cells is not sufficient to link ch-TOG to the majority of the EB1 pool, unlike in Swiss 3T3 and CHO cells [24], thus enabling the specific functions of EB1 and ch-TOG to be analysed in HeLa cells.

### Independent Actions of EB1 and ch-TOG on Microtubule Dynamicity in Interphase HeLa Cells

We next compared the impact of depleting EB1 and/or ch-TOG by RNAi on microtubule dynamics (Figure S2E, S6F). Because dynamic instability parameters following depletion of EB1 or XMAP215/TOG family proteins have been reported in many independent studies [5], [6], [16], [24], [36], [37], and because we observed similar tendencies, in this study we sought to compare their actions on the overall dynamicity of microtubules. Consistent with previous observations in EB1- or ch-TOG-depleted cells, the lifetime of individual microtubules was obviously increased (Figure 8A–E; Movie S5). Statistical analysis of the “pause” state, in which microtubules exhibit no detectable growth/shortening or repeated growth/shortening over a limited distance (<1 µm), demonstrated that the pause duration was increased in cells in which either EB1 or ch-TOG was depleted, as expected, and further increased in cells in which both EB1 and ch-TOG were depleted (Figure 8F). We also performed western blotting analyses using an antibody against acetylated tubulin, a marker of stable microtubules (Figure 8G, 8H, S6A). We repeatedly observed the same trends in changes in the proportion of acetylated tubulin, but statistical significance was not reached for some data because of the small sample size. Consistent with previous observations, depletion of ch-TOG increased the stability of microtubules. Interestingly, the microtubule stability was also increased after depletion of EB1. This result is consistent with previous *in vitro* studies, which demonstrated that EB1 can increase the dynamicity of microtubules [5], [15]. When EB1 and ch-TOG were depleted simultaneously, a greater effect on microtubule stability was observed, suggesting their independent and complementary activities. All of these results indicate that both EB1 and ch-TOG by themselves stimulate the dynamic growth/shortening of pausing microtubule ends to increase microtubule dynamicity.

Previously, it has been reported that EB3, another EB1 family protein, but not EB2, has similar activity in regulating microtubule dynamics [5], [38]. To examine the contribution of EB3, we measured the relative expression levels of EB proteins in HeLa cells (Figure S5, S7D). Although *EB3* gene expression in HeLa cells could indeed be detected by RT-PCR (data not shown), the expression level of EB3 in HeLa cells was very low; it was calibrated to be less than 0.6% of endogenous EB1. Considering that ∼12% of endogenous EB1 remained after siRNA-mediated knockdown (Figure S2E, S6F), the amount of EB3 in EB1-delpeted cells is minimal and therefore the effect of EB3 would be negligible.

### Microtubule-anchoring Function of EB1 in Interphase HeLa Cells

Finally, we investigated the organisation of microtubules. Although microtubule organisation did not change significantly in ch-TOG-depleted cells, pronounced alterations in microtubule patterns were observed when EB1 was depleted; i.e., the microtubule density was reduced significantly at the periphery of the cells (Figure 9A). A similar phenotype was observed when CLASPs were depleted, as we reported previously [23]. CLASPs bind to microtubules and EB1, as well as to the PH-domain-containing cortical proteins LL5α and LL5β [31]–[33], and thereby attach microtubule ends to the basal cell cortex (Figure 9D). EB1 also binds to other cortex binding +TIPs that have the ability to anchor microtubules, such as the ACF7 and APC tumour suppressor proteins [39]–[43]. Therefore, loss of EB1 function is considered to reduce microtubule association with the basal cell cortex, but this hypothesis has never been tested. To examine this hypothesis, we used TIRF microscopy, which illuminates only a very shallow depth (∼200 nm) at the bottom of the cell, and thus enables the study of cortical microtubule attachment [23], [44], [45]. In sharp contrast to control cells in which many microtubule ends are visualised near the cell periphery, no obvious peripheral accumulation of microtubule ends was observed in EB1-depleted cells, while ch-TOG-depletion did not affect the microtubule distribution at the basal cell cortex (Figure 9B, C). Expression of mouse EB1 (mEB1) as a rescue construct (Figure S2G, S7B) restored the peripheral microtubule density. In addition, we also examined whether EB3 has microtubule-anchoring activity. In cells expressing exogenous EB3 (Figure S2H, S7C), the microtubule density was retained at the basal cell cortex after EB1-depletion, demonstrating that EB3 has a similar function to EB1 in anchoring microtubules to the cell cortex.

CLASPs bind to EB1 and are potent microtubule-anchoring factors in HeLa cells, as we reported previously [23]. When GFP-CLASP2γ was overexpressed in HeLa cells, endogenous EB1 was strongly recruited to the CLASP-concentrated microtubule ends, whereas the distribution of ch-TOG was not affected (Figure 10A). Comparison of the distributions of mRFP1-CLASP2γ and ch-TOG at higher resolution revealed that ch-TOG was associated with the tips of CLASP-decorated microtubule ends (Figure 10B–D). Thus, the linkage between the EB1-CLASP complex and the cortex should still allow tubulin addition at the very ends of the microtubules, possibly delivered by ch-TOG (Figure 9D).

## Discussion

In this study, we revealed for the first time the different localisations of EB1 and ch-TOG at the plus ends of microtubules in cells using high-resolution microscopy techniques, and confirmed their non-overlapping binding to the plus ends of microtubules by overexpression studies. Obviously, the microtubule-end-recognition mechanism is different for EB1 and ch-TOG. Our observations demonstrated that the ch-TOG accumulation sites protrude ∼100 nm from the EB1 comets. This finding suggests the presence of an unexpectedly long extended structure that is not recognised by EB1 at the end of newly polymerised microtubule ends. Despite the numerous *in vitro* studies that have investigated the structure of growing microtubule ends using purified tubulin, their dynamic configuration in living organisms is still largely unknown. Therefore, our findings in this study provide new insights into the dynamic structure of polymerising microtubule ends.

Ch-TOG has been proposed to bind either to single tubulin dimers [3] or to small tubulin oligomers [46] at its NH_2_-terminus and add them to microtubule ends. The localisation of ch-TOG at the microtubule tip is consistent with either of these models. In contrast, EB1 was very recently demonstrated to contact four different tubulin dimers in the B-lattice [12], in which the α-tubulins of one protofilament are arranged next to α-tubulins in the neighbouring protofilaments (Figure 11). This means that EB1 binds to microtubules following the lateral association of the protofilaments, which occurs after tubulin polymerisation at their ends. Maurer et al. have predicted that EB1 would not bind at the very ends of growing microtubules owing to incomplete formation of their binding sites. As predicted, we observed GFP-α-tubulin signals in front of EB1 comets in SIM images. This is the first instance of the detection of microtubule signals ahead of EB1 comets. Because the GFP-α-tubulin signals decay gradually from the peak position of the EB1 comet towards the distal end, the microtubule tip is thought to contain protofilaments of different lengths (Figure 11). Probably only a subset of protofilaments positive for ch-TOG grows faster. Our analyses suggested that, by using the unique distributions of ch-TOG as a reference, the frayed protofilaments can elongate more than 100 nm. The 100 nm protofilament is quite long, and it contains ∼12 tubulin dimers (8 nm in the long axis). The probable presence of long, frayed protofilaments has been predicted by the mechanochemical model of microtubule structure and self-assembly kinetics proposed by VanBuren *et al.* [47]. Their simulation of microtubule assembly showed that XMAP215 promotes the formation of long, curved protofilament extensions. Our observations in this study therefore fit well with previous models proposed in independent studies, and provide evidence of the extent of protofilament extension during microtubule growth in cells.

Second, we demonstrated that both EB1 and ch-TOG had similar effects in increasing microtubule dynamicity, but only EB1 exhibited microtubule-anchoring activity at the basal cell cortex. We showed that the function of EB3 was redundant. Following the previous findings of a conserved EB1-recognition mechanism involving an SxIP motif and its importance in epithelial cell morphogenesis [20], [21], our findings here conclusively demonstrated that EB1 family proteins provide a functional core that mediates the anchoring of microtubules to the cortex. EB1 binds to CLASPs, which are potent microtubule-anchoring factors at the basal cell cortex [23], as well as other cortical factors such as ACF7 and APC [42], [43]. Therefore, the loss of the linkage between EB1 and these cortex +TIPs could be the major cause of the reduced microtubule-cortex attachment observed after EB1-depletion. Previously, we observed that microtubules attached to the cell cortex by CLASPs remain dynamic [23]. The density of microtubule ends is maintained at the CLASP-accumulating cell cortex, but individual microtubules continue their repetitive growth and shortening over a very short distance, while free microtubule ends exhibit longer growth/shortening episodes. At the microtubule-attachment sites, both the catastrophe and rescue frequencies are increased such that the length of microtubules can be maintained for certain periods of time. Our observations here explain these phenomena because ch-TOG, a potent anti-pause factor, can still access the very ends of anchored microtubules. Because dynein and kinesin motor proteins also bind to the ridge of single protofilaments [48], EB1-mediated microtubule anchoring may allow the recruitment of other microtubule modulators such as the microtubule destabilising kinesin, MCAK [49], or cargo loading via motor proteins at the very ends of microtubules.

Recent reports demonstrated that microtubule capture mediated by CLASP or APC is important for maintaining a normal neuromuscular phenotype in mouse muscles or convergent extension cell movements in developing *Xenopus* oocytes, respectively [50], [51]. In plants, CLASP localisation is required for the parallel ordering of microtubules at the cortex to define the direction of cell expansion [52]. EB1 family proteins may be involved in these microtubule-anchoring processes, which are necessary for organ function and development. The microtubule-anchoring function of EB1 family proteins has been observed in yeast, in which EB1-mediated attachment of microtubule ends to the growing tips helps to deliver cargos such as nuclear and cell polarity molecules [reviewed 53,54], indicating the evolutionarily-conserved role of EB1 family proteins. Therefore, microtubule-anchoring activity, rather than the regulation of microtubule dynamics that can be performed by various other molecules, may be the crucial function of EB1 family proteins.

## Materials and Methods

### Expression Constructs

The pEGFP-C and -N series plasmids (TaKaRa/Clontech), pEGFP-tubulin (TaKaRa/Clontech), pTagRFP-tubulin (evrogen) and pTagRFP-EB3 (evrogen) plasmids were obtained from the indicated sources. TagRFP was used after introducing the S162T mutation (TagRFP-T; referred to simply as RFP in this report) to improve photostability [22]. The pTagRFP-T-C and -N series plasmids were generated by substituting the EGFP moiety of the pEGFP-C and -N series plasmids with TagRFP-T. Cloning of the mouse EB1 cDNA (mEB1) and GFP-fused mEB1 (mEB1-GFP) expression constructs was described previously [11]. TagRFP-T-fused mEB1 (mEB1-RFP) was generated by substituting EB3 cDNA in the pTagRFP-T-EB3 vector for mEB1 cDNA. Human EB1 and EB2 cDNAs were PCR-cloned using first-strand cDNA generated from HeLa cells. Human EB1, EB2 and EB3 cDNAs were inserted into the NheI and HindIII sites of pEGFP-N1. Human ch-TOG cDNA (BC120869 MGC:134926 IMAGE:40072423) was obtained from Open Biosystems. To fuse GFP to the NH_2_-terminus or the COOH-terminus of ch-TOG, the ch-TOG cDNA was inserted into the BglII and BamHI sites of pEGFP-C2 (GFP-ch-TOG), or the KpnI and BamHI sites of pEGFP-N3 (ch-TOG-GFP), using the In-Fusion HD Cloning Kit (TaKaRa/Clontech). GFP-CLASP2γ, mRFP1-CLASP2γ and GFP-SLAIN2 have been described previously [23], [24]. For ecotropic retrovirus-mediated gene transfer, cDNAs were inserted into pCX4 series retroviral vectors carrying different drug-selection markers [25]. The GFP-α-tubulin moiety from the pEGFP-tubulin plasmid was inserted into pCX4hyg at the BamHI and NotI sites. The TagRFP-T-α-tubulin moiety from the pTagRFP-T-tubulin plasmid was inserted into pCX4pur at the EcoRI and MulI sites using the In-Fusion HD Cloning Kit. The mEB1 cDNA was inserted into pCX4neo at the NotI and HpaI sites. For lentivirus-mediated gene transfer, the PGK promoter and the neomycin resistance gene sequences of the pLVSIN-EF1α Neo plasmid (TaKaRa) were substituted for the IRES-puromycin resistance gene sequence from the pCX4pur plasmid or the IRES-neomycin resistance gene sequence from the pCX4neo plasmid to generate pLVSIN-EF1α-IRESpur or pLVSIN-EF1α-IRESneo, respectively. The human EB3 cDNA with 5′ EcoRI and 3′ EcoRV sites was inserted into the EcoRI and HpaI sites of pLVSIN-EF1α-IRESneo. The mEB1-TagRFP-T cDNA with 5′ BglII and 3′ NotI sites was inserted into the BamHI and NotI sites of the pLVSIN-EF1α-IRESpur plasmid. This plasmid was also used to transiently overexpress mEB1-RFP under the control of the EF1α promoter in cells expressing GFP-fusion proteins under the control of the CMV promoter.

### Cells and Gene Transfer

Human HeLa cells [23] and Cos7 cells (Health Science Research Resources Bank) were maintained in Dulbecco’s modified Eagle’s medium (DMEM; Gibco) supplemented with 10% foetal bovine serum (FBS), 100 U/ml penicillin and 100 µg/ml streptomycin at 37°C under a 5% CO_2_ atmosphere. Plasmid transfections were performed using the Effectene transfection reagent (Qiagen) according to the manufacturer’s instructions. Retroviral-mediated gene transfer was performed as described previously [26]. Briefly, HeLa cells were first transiently transfected with the murine ecotropic retrovirus receptor (mCAT1), which renders HeLa cells susceptible to subsequent infection with ecotropic viral vectors [25]. The cDNAs were inserted into pCX4 series retroviral vectors carrying different drug-selection markers [25] and retroviruses were generated in HEK293T Lenti-X cells (TaKaRa) using a Retrovirus Packaging Kit Eco (TaKaRa). Lentiviral-mediated gene transfer was performed according to the manufacturers’ instructions using a pLVSIN-EF1α (TaKaRa)-based plasmid, as described above. Lentiviruses were generated in HEK293T Lenti-X cells (TaKaRa) using the Lenti-X HTX Packaging System (TaKaRa). The infected HeLa cells were selected in medium containing appropriate drugs. To obtain cell populations expressing sufficient and equal amounts of GFP-α-tubulin or RFP-α-tubulin, transfected cells were cloned by single cell sorting using FACSVantage SE with Diva option (BD). Clone 1E10 for GFP-α-tubulin and clone 1A2 for RFP-α-tubulin were selected for use in this study.

For immunostaining, cells were seeded at a density of 3–5×10^4^ cells/cm^2^ on uncoated No.1S coverslips (Matsunami) or coverslips coated with collagen using Collagen Coating Solution (TOYOBO). For live cell imaging, cells were seeded on collagen-coated 35-mm glass-based dishes (No. 1S coverslip, IWAKI) at a density of 3–5×10^4^ cells/cm^2^ and observed in DMEM^gfp^ medium (evrogen).

### Transfection of siRNAs

Synthetic stealth siRNAs (Invitrogen) were transfected using the HiPerFect transfection reagent (Qiagen) according to the manufacturer’s instructions. siRNAs were directed against the following target sites: human EB1, GGTCAACGTATTGAAACTTACTGTT; human ch-TOG, TGAAAAGAGCCCAGAGTGGTCCAAA (the established siRNA sequence against human ch-TOG [27] was converted to the stealth siRNA format). The siRNAs were transfected at a concentration of 100 nM. The knockdown efficiency was analysed by western blotting. Because ch-TOG depletion inhibits mitosis progression and induces cell death, siRNA-treated cells were blocked in interphase 1 d after transfection by adding 2.5 mM thymidine (Sigma-Aldrich) to the culture medium for 2 d. The cells were analysed 3 d after siRNA transfection.

### Antibodies

Anti-human ch-TOG rabbit polyclonal antibody (pAb) was raised against amino acids 844–1,972 of human ch-TOG and has been described previously [28]. Anti-LL5β rabbit pAb has been described previously [29]. The following primary antibodies were purchased from the indicated sources: mouse anti-EB1 monoclonal antibody (mAb) (clone 5, BD Transduction Laboratories); rabbit anti-EB1 pAb (Abcam; this antibody weakly reacts with tubulin.); rabbit anti-EB2 pAb (Abcam); rabbit anti-EB3 pAb (Abcam); rat anti-CLASP1 mAb (KT66, absea); rat anti-CLASP2 mAb (KT69, absea); mouse anti-α-tubulin mAb (DM1A, Sigma); FITC-conjugated mouse anti-α-tubulin mAb (DM1A, Sigma); rat anti-α-tubulin mAb (YL1/2, Abcam); rabbit anti-β-tubulin pAb (Abcam); rabbit anti-GFP pAb (Chemicon); rabbit anti-TagRFP antibody (evrogen). As secondary antibodies, DyLight 405-, Cy2-, Rhodamine Red-X (RhoX)-, DyLight 649 (DL649)- and HRP-conjugated anti-mouse, anti-rabbit and anti-rat antibodies for multi-labelling (ML) were purchased from Jackson ImmunoResearch.

### Western Blotting

Total cell lysates were separated by SDS-PAGE and transferred to PVDF membranes using the mini-PROTEAN system (Bio-Rad), and probed with the appropriate antibodies. Proteins were visualised with ECL Plus Western Blotting Detection Reagents (Amersham), ECL Select Western Blotting Detection Reagents (Amersham) or Chemi-Lumi One Super reagent (Nacalai Tesque) and detected using a LuminoImager (LAS-3000, FujiFilm) or a Davinhci-Chemi (CoreBio).

### Immunofluorescence Staining

For conventional two-dimensional culture, the cells were seeded onto uncoated or collagen-coated coverslips and the culture medium was exchanged 1–2 h before fixation [23]. The cells were fixed in a mixture of acetone:methanol (1∶1) at –20°C for 5 min. The coverslips were directly transferred from culture medium at 37°C to a large pool of the fixing solution at –20°C. The fixed specimens were washed twice in phosphate-buffered saline (PBS) containing 0.01% Triton X-100 and incubated in 10% FBS. For immunostaining, primary antibodies were diluted in Can Get Signal Immunostain Solution B (TOYOBO) as indicated below: anti-ch-TOG pAb, 1∶1000; anti-EB1 mAb, 1∶100; anti-EB1 pAb, 1∶500; FITC-conjugated or unlabelled anti-α-tubulin mAb DM1A, 1∶50; anti-α-tubulin mAb YL1/2, 1∶50; anti-LL5β pAm, 1∶1000. The specimens were incubated with primary antibodies for 1 h and washed three times in PBS, and then incubated with secondary antibodies diluted 1∶100 for anti-tubulin antibodies and 1∶200 for other antibodies in Can Get Signal Immunostain Solution B for 1 h. After washing three times in PBS, the samples were mounted in ProLong Antifade Reagent (Molecular Probes).

### Fluorescence Microscopy and Image Analysis

Confocal imaging was performed using an LSM780 laser scanning confocal microscope equipped with an Axio Observer.Z1 inverted microscope with a Plan-APOCHROMAT 63×/1.40 NA oil immersion objective, four laser lines (405 nm; Multi-Ar, 458, 488, 514 nm; DPSS, 561 nm; He-Ne, 633 nm), and a highly-sensitive Gallium Arsenide Phosphide (GaAsP) detector (Carl Zeiss). Wide-field epi-fluorescence live cell imaging was performed using a DeltaVision Core microscope system (Applied Precision Inc.) equipped with an IX71 inverted microscope and PlanApo N 60×/1.42 NA and UPlanApo 100×/1.35 NA oil immersion objectives (Olympus), a mercury lamp light source and a stage top CO_2_ incubator (Tokken). The collected images were subjected to deconvolution calculations using software installed on this system. The TIRF microscope was composed of a Ti-E PFS inverted microscope equipped with a Perfect Focus autofocus system, CFI Apo TIRF 100×/1.49 NA oil immersion objective (Nikon), two diode laser lines (488 nm, Coherent; and 561 nm, Melles Griot), W-view optics (Hamamatsu), an EMCCD camera iXonEM+ DU897E-CSO-#BV (Andor), and MetaMorph control software (Molecular Devices).

SIM was performed using an ELYRA PS.1 equipped with an Axio Observer.Z1 inverted microscope with an α Plan-APOCHROMAT 100×/1.46 NA oil immersion objective (Carl Zeiss), four diode laser lines (405, 488, 561, 642 nm), an EMCCD camera iXon885 (Andor), and system control software ZEN2010 C or ZEN2010 D. Z-stacks of 20–25 serial optical sections were collected at 84-nm intervals with three rotation of gratings. SIM images were reconstructed using software installed on the ELYRA PS.1. This system often generates abnormal SIM images containing unexpected residual intensities around objects, which can be recognised when observing well-established patterns such as EB1 comets and microtubule filaments. These artefacts may be attributed to drift of the specimen, changes in illumination/detection intensities, or bleaching properties of the sample during acquisition [30]. To obtain appropriate SIM images without apparent artefacts, >20 data sets were routinely collected for each experiment and images containing abnormal signals were omitted from further processing. Then, the pixel shift and chromatic aberration in the multicolour SIM images were corrected using a reference beads image. All measurements were performed on single z-sections close to the coverslips. The derived SIM images contain 25×25 nm pixel pitch spatial information.

The distribution of proteins along microtubules was measured by line profile analysis of fluorescence intensity using ImageJ software. To average multiple line profiles, each data set was aligned at the peak position of EB1 because the shape of EB1 is relatively uniform. The average line profiles were generated using multiple SIM images to avoid possible bias from certain artefacts included in each SIM image. When analysing ch-TOG distribution in cells overexpressing mEB1-GFP, in which mEB1-GFP distributes throughout the entire microtubule lattice (Figure 6C), the line profiles were aligned at the peak position of the ch-TOG signal found at the microtubule ends. When the data set contained only ch-TOG and GFP-α-tubulin signals (Figure 3I), the line profiles were aligned at the peak position of ch-TOG staining in the Rhodamine Red-X channel. To analyse statistical significance between the ch-TOG distributions in GFP-SLAIN2-expressing cells and control cells (Figure 7F), after aligning each ch-TOG line profile at the EB1 peak position the intensity was normalized to the value of the peak closest to the EB1 peak, and then the data sets at same relative position in these two cells were analysed with two-sample *t*-test. The peak-to-peak separation between EB1 and ch-TOG clusters labelled with two different fluorophores was obtained by detecting the peak positions in the line profiles at 25 nm resolution. The ch-TOG signals often contained multiple peaks in the line profile. We selected a peak closest to the EB1 peak, not the highest peak, between –100 and 300 nm from the EB1 peak. Data containing no obvious ch-TOG peak were used to calculate average line profiles but were omitted from measurements of peak-to-peak separation. This procedure overcomes the limitation of resolution in the SIM system analogous to a method developed by Wan et al. [31], in which the average label separation was measured by determining the centroids of protein clusters by standard nonlinear Gaussian curve fitting that achieved < 5-nm accuracy in wide-field epi-fluorescence images.

Fluorescent signals were quantified and analysed using ImageJ software or MetaMorph software (Molecular Devices). Colocalisation analysis was performed using IMARIS software (Andor/Bitplane). Microtubule dynamics were analysed using time-lapse images collected with a DeltaVision system, as described previously [5], [23], [24]. The statistical significance of the differences was assessed using a two-sample *t*-test or Mann-Whitney U test. Movies were generated using softWoRx installed on the DeltaVision system, MetaMorph and ImageJ software. To reduce noise in the TIRF images, the walking average function (3 frames average) was used.

## Supporting Information

Figure S1 Distribution of microtubules and CLASP-LL5 complexes in HeLa cells cultured on uncoated or collagen-coated coverslips.
(DOC)

Figure S2 Characterisation of HeLa cell transfectants and siRNA tools.
(DOC)

Figure S3 Visualisation of the very ends of microtubules.
(DOC)

Figure S4 Comparison and evaluation of the ch-TOG GFP-fusion constructs.
(DOC)

Figure S5 Measurement of the relative expression levels of EB1, EB2 and EB3 in HeLa cells.
(DOC)

Figure S6 Full scan western blots 1.
(DOC)

Figure S7 Full scan western blots 2.
(DOC)

Movie S1 Behaviour of ch-TOG-GFP and mEB1-RFP in a living HeLa cell visualised by TIRF microscopy. The image sequence corresponds to Figure 4A. In the right, the ch-TOG-GFP (red) and mEB1-RFP (green) signals are merged. Scale bar, 10 µm; time scale, s:ms.
(MOV)

Movie S2 Behaviour of ch-TOG-GFP and mEB1-RFP in a living HeLa cell visualized by TIRF microscopy (averaged). The image sequence corresponding to Movie S1 was processed in ImageJ using the ‘WalkingAverage’ plugin set to a 3-frames average to reduce image noise. This allows better visualization of small ch-TOG spots. Note, however, that in this movie the temporal resolution is reduced. Scale bar, 10 µm; time scale, s:ms.
(MOV)

Movie S3 Behaviour of ch-TOG-GFP and RFP-α-tubulin in a living HeLa cell visualised by TIRF microscopy. The image sequence corresponds to Figure 4C. In the right, the ch-TOG-GFP (red) and RFP-α-tubulin (green) signals are merged. Time lapse images of the boxed area are shown in Fig. 1h. Scale bar, 5 µm; time scale, s:ms.
(MOV)

Movie S4 Behaviour of ch-TOG-GFP and RFP-α-tubulin in a living HeLa cell visualized by TIRF microscopy (averaged). The image sequence corresponding to Movie S3 was processed in ImageJ using the ‘WalkingAverage’ plugin set to a 3-frames average to reduce image noise. This allows better visualization of small ch-TOG spots. Note, however, that in this movie the temporal resolution is reduced. Scale bar, 10 µm; time scale, s:ms.
(MOV)

Movie S5 Impact of EB1 and/or ch-TOG depletion on microtubule dynamics. The image sequence corresponds to Figure 8A–D. HeLa/GFP-α-tubulin clones (1E10) were treated with the indicated siRNAs and imaged with a 0.5 s interval using the DeltaVision system. Scale bar, 10 m; time scale, h:m:s:ms.
(MOV)

Text S1 Supplemental Results and Discussion.
(DOC)
