# Supplementary material for: Dissecting the Nanoscale Distributions and Functions of Microtubule-End-Binding Proteins EB1 and ch-TOG in Interphase HeLa Cells
Source: PLoS One. 2012 Dec 12;7(12):e51442. doi: 10.1371/journal.pone.0051442 (PMC3520847; doi:10.1371/journal.pone.0051442)
Supplement: Figure S1 — Distribution of microtubules and CLASP-LL5 complexes in HeLa cells cultured on uncoated or collagen-coated coverslips. (DOC) [file pone.0051442.s001.doc]

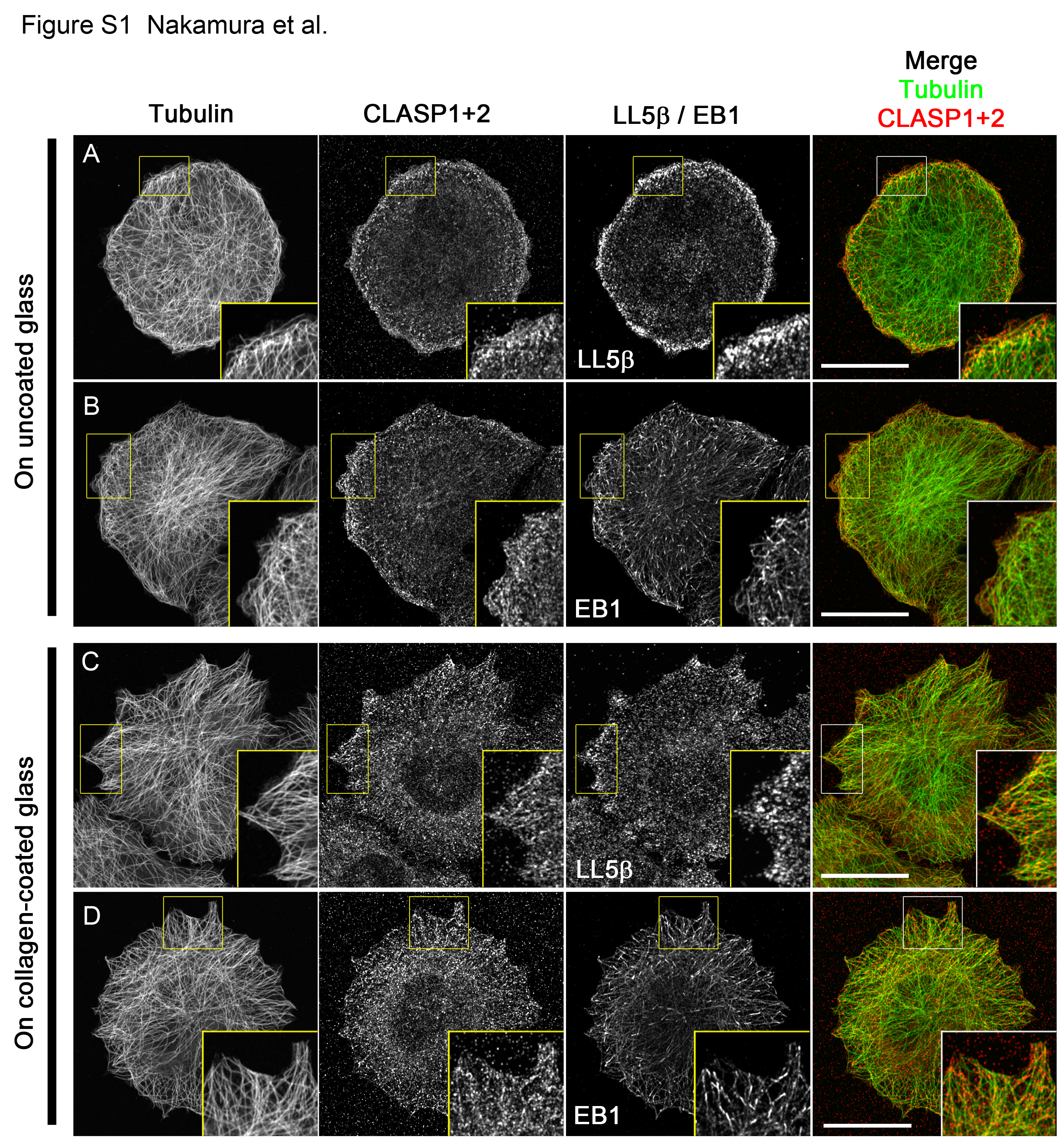


**Figure S1.**

**Distribution of microtubules and CLASP-LL5 complexes in HeLa cells cultured on uncoated or collagen-coated coverslips.** HeLa cells cultured on uncoated coverslips (**A**, **B**) or collagen-coated coverslips (**C**, **D**) were fixed and immunostained as indicated in the figure. Images were acquired by confocal microscopy. To immunostain CLASP1 and CLASP2 simultaneously (CLASP1+2), anti-CLASP1 and anti-CLASP2 antibodies were mixed. In the panels on the right, tubulin signals (green) and CLASP1+2 signals (red) are merged. The insets show the boxed areas at 2× magnification. Note that in cells seeded onto uncoated coverslips microtubules and EB1 are densely accumulated at the cell edges where CLASPs and LL5β are obviously clustered (A, B), while on collagen-coated coverslips CLASPs and LL5β were observed to be distributed more diffusely near the cell periphery (C, D). Because the peripheral microtubules on uncoated coverslips were too dense to enable the visualisation and analysis of individual filaments separately at high resolution, we cultured cells on collagen-coated glass in this study. For a more detailed description, see Text S1. Scale bars, 20 μm.
