# Supplementary material for: Dissecting the Nanoscale Distributions and Functions of Microtubule-End-Binding Proteins EB1 and ch-TOG in Interphase HeLa Cells
Source: PLoS One. 2012 Dec 12;7(12):e51442. doi: 10.1371/journal.pone.0051442 (PMC3520847; doi:10.1371/journal.pone.0051442)
Supplement: Figure S2 — Characterisation of HeLa cell transfectants and siRNA tools. (DOC) [file pone.0051442.s002.doc]

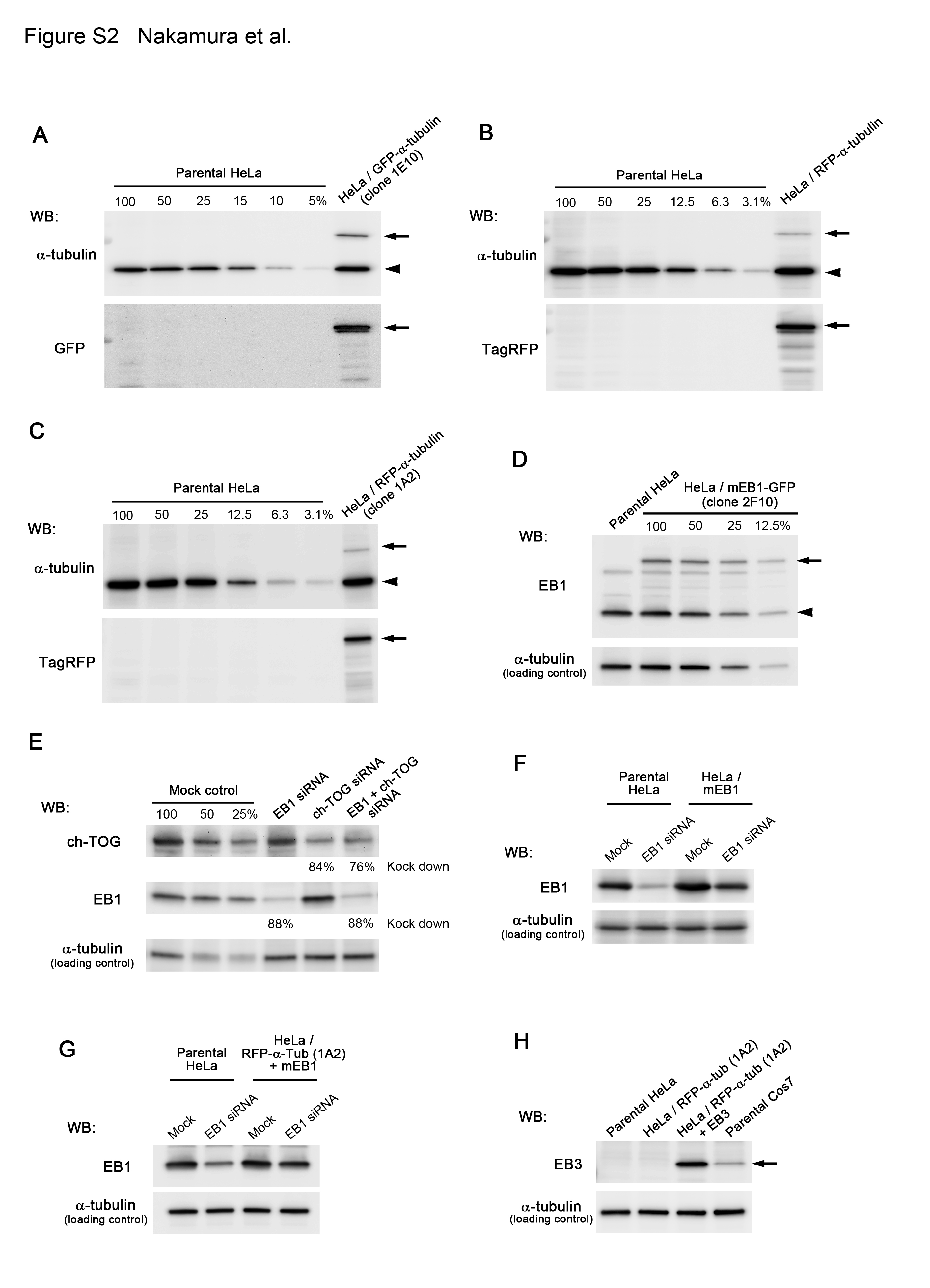
**Figure S2.**

**Characterisation of HeLa cell transfectants and siRNA tools.** (**A**) Establishment of the GFP-α-tubulin expressing cell line. HeLa cells expressing GFP-α-tubulin (clone 1E10) were lysed and subjected to western blotting (WB), for which a dilution series of parental HeLa cell lysate was used to calibrate the expression level of GFP-α-tubulin. The undiluted lysates were loaded at 5 μg per lane. The expression level of GFP-α-tubulin (arrow) was estimated to be ~12% of that of endogenous α-tubulin. (**B**) Establishment of RFP-α-tubulin expressing HeLa cells (uncloned). The cells lysates were subjected to WB in the same manner as GFP-α-tubulin. The average expression level of RFP-α-tubulin (arrow) was estimated to be ~4% of that of endogenous α-tubulin. In the imaging experiments, the cells with higher expression levels were observed. (**C**) Establishment of the RFP-α-tubulin expressing HeLa cell line (clone 1A2). The expression level of RFP-α-tubulin was estimated to be ~4% of that of endogenous α-tubulin. (**D**) Establishment of the mEB1-GFP expressing HeLa cell line (clone 2F10). The expression level of mEB1-GFP was estimated to be ~40% of that of endogenous EB1. (**E**) Validation of the knockdown efficiency of siRNAs against human EB1 and ch-TOG. Lysates of cells treated with the indicated siRNAs were subjected to WB with the indicated antibodies. A dilution series of the mock-transfected sample was used to create a standard curve and the calibrated density was normalised to the α-tubulin signals. The knockdown efficiency is indicated at the bottom of the each band. (**F**) Establishment of mEB1 expressing HeLa cells. The mEB1 construct was used for rescue experiments in this study. HeLa cells expressing mEB1 (uncloned) were treated with an siRNA against human EB1 and subjected to WB using the indicated antibodies. Although the band for exogenous mEB1 cannot be separated from the endogenous EB1 band, the EB1 band remained at a level roughly equal to endogenous EB1 after depletion of EB1 with an siRNA against human EB1. (**G**) Introduction of mEB1 into the RFP-α-tubulin expressing HeLa cell line (clone 1A2). Expression of mEB1 was confirmed by depleting endogenous EB1 as in (F). (**H**) Introduction of EB3 into the RFP-α-tubulin expressing HeLa cell line (clone 1A2). A Cos7 cell lysate was used as a positive control for EB3 detection, because endogenous EB3 in HeLa cells is undetectable by WB. In (A), (B), (C), (D), (G) and (H), endogenous and exogenous proteins are indicated by the arrowheads and arrows, respectively. For WB full scans, see also Figure S6B-F, S7A-C.
