# Supplementary material for: Dissecting the Nanoscale Distributions and Functions of Microtubule-End-Binding Proteins EB1 and ch-TOG in Interphase HeLa Cells
Source: PLoS One. 2012 Dec 12;7(12):e51442. doi: 10.1371/journal.pone.0051442 (PMC3520847; doi:10.1371/journal.pone.0051442)
Supplement: Figure S3 — Visualisation of the very ends of microtubules. (DOC) [file pone.0051442.s003.doc]

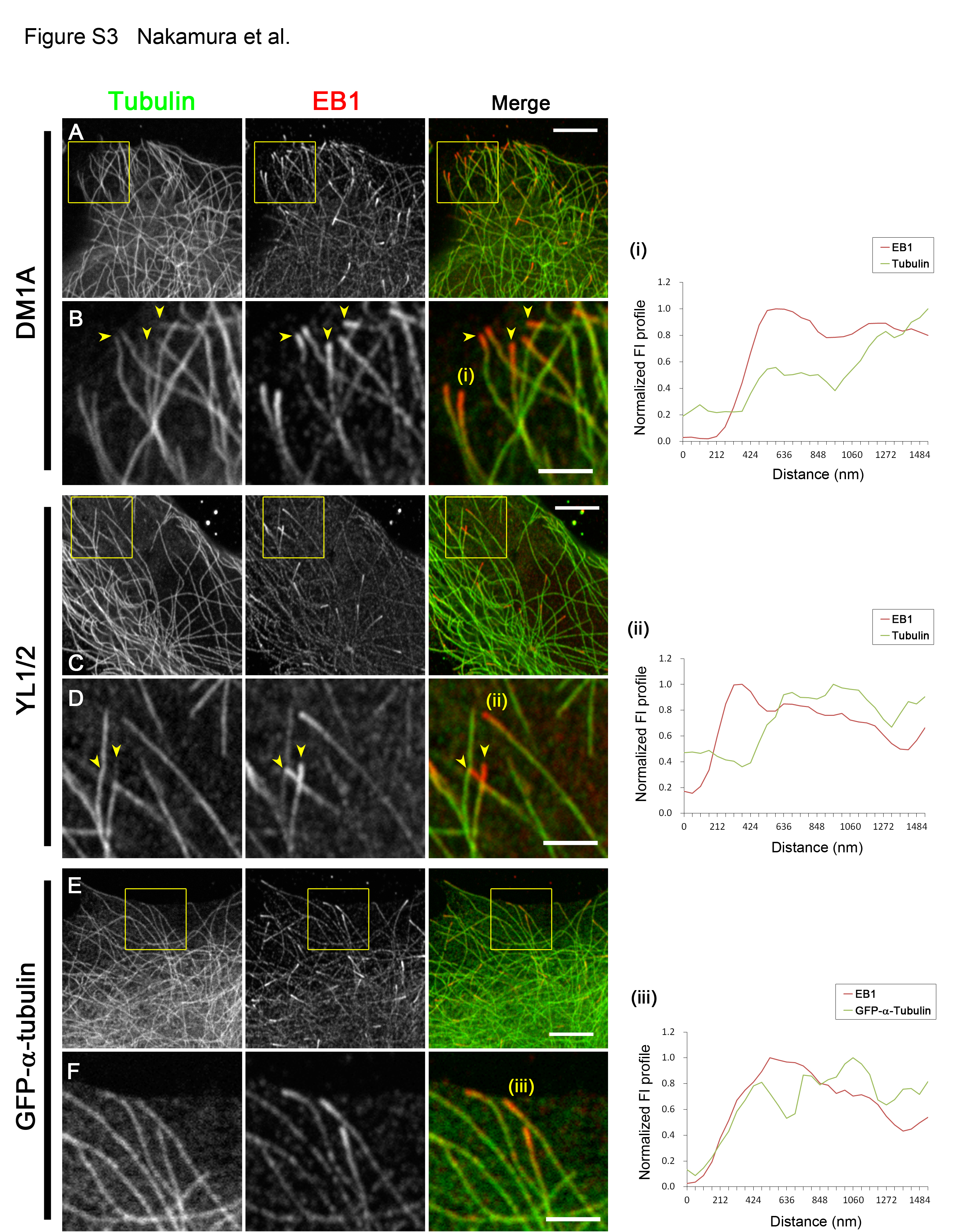


**Figure S3.**

**Visualisation of the very ends of microtubules.** HeLa cells cultured on collagen-coated coverslips were fixed and stained for tubulin (with either FITC-conjugated DM1A (**A**, **B**) or YL1/2 (**C**, **D**) anti-α-tubulin antibodies) and EB1, or HeLa cells expressing GFP-α-tubulin (clone 1E10) were fixed and stained for EB1 (**E**, **F**). (B), (D) and (F) are magnified images of the boxed areas. Positions of microtubule ends visualised by EB1 signals but showing weak microtubule staining are indicated by the arrowheads. Line profiles along the microtubules indicated by (i) – (iii) are shown on the right. For a more detailed description, see Text S1. Scale bars, 5 μm (A, C, E); 2 μm (B, D, F).
