# Supplementary material for: Dissecting the Nanoscale Distributions and Functions of Microtubule-End-Binding Proteins EB1 and ch-TOG in Interphase HeLa Cells
Source: PLoS One. 2012 Dec 12;7(12):e51442. doi: 10.1371/journal.pone.0051442 (PMC3520847; doi:10.1371/journal.pone.0051442)
Supplement: Figure S4 — Comparison and evaluation of the ch-TOG GFP-fusion constructs. (DOC) [file pone.0051442.s004.doc]

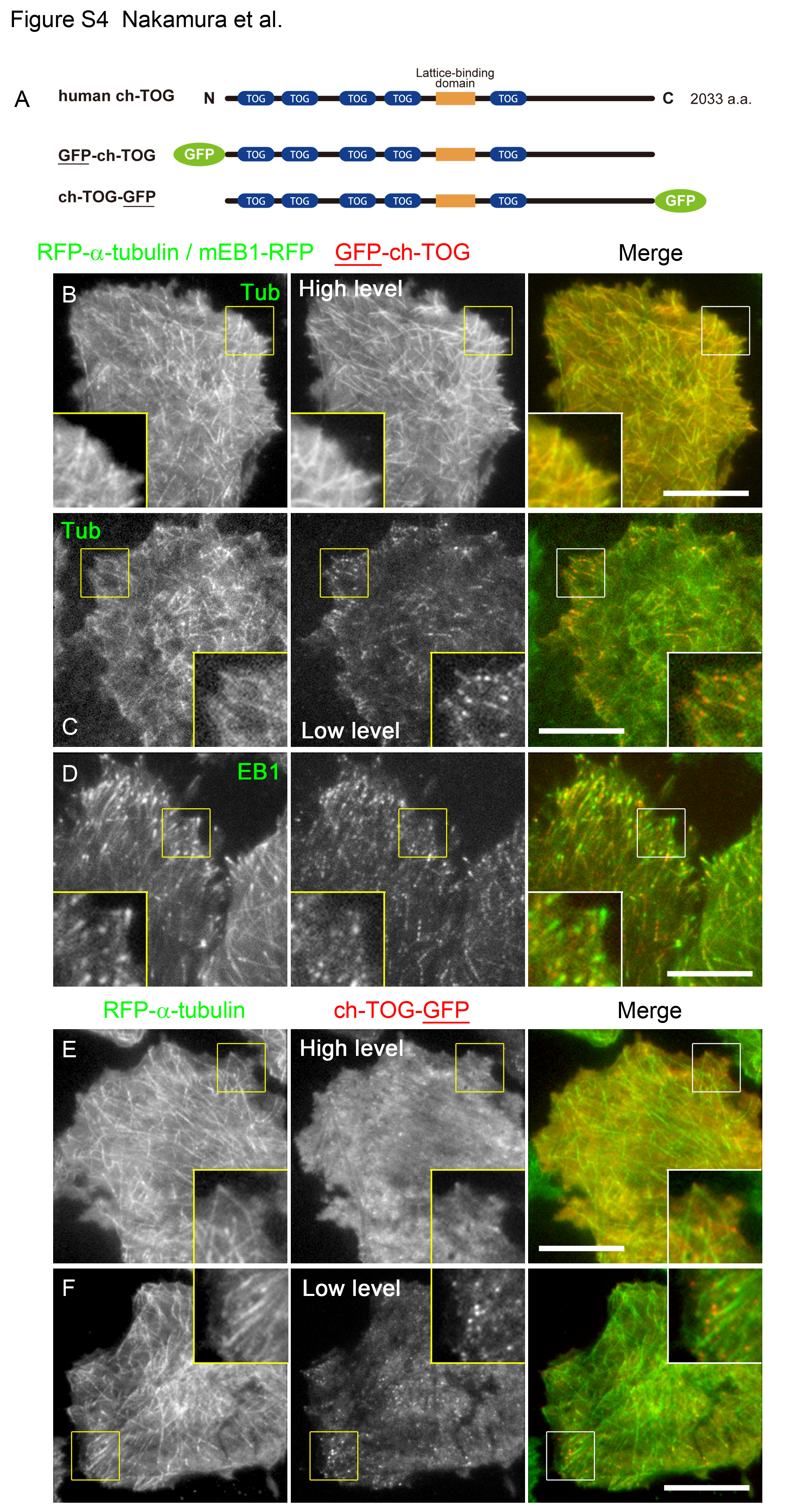


**Figure S4.**

**Comparison and evaluation of the ch-TOG GFP-fusion constructs.** (**A**) Domain structures of the ch-TOG and GFP-fused ch-TOG constructs used in this study. This domain structure is conserved in vertebrate orthologues of ch-TOG, including *Xenopus laevis* XMAP215, and also in *Drosophila melanogaster* Mini Spindles (Msps). GFP was fused to the NH_2_-terminus (GFP-ch-TOG) or COOH-terminus (ch-TOG-GFP) of ch-TOG. RFP-α-tubulin or mEB1-RFP and GFP-ch-TOG (**B-D**), or RFP-α-tubulin and ch-TOG-GFP (**E**, **F**), were expressed in HeLa cells and observed by TIRF microscopy. In the merged panels on the right, RFP and GFP signals are shown in green and red, respectively. For each set of transfectants with RFP-α-tubulin, cells with a high (**B, E**) or low (**C**, **F**) level of ch-TOG expression are shown. Note that GFP-ch-TOG strongly distributes throughout the microtubule lattice when expressed at a high level, while ch-TOG-GFP binds only very weakly to the microtubule lattice even when expressed at a high level. Unlike ch-TOG-GFP, which distributes to the majority of EB1 comets (Figure 4A), GFP-ch-TOG dots were detectable only at a subset of EB1 comet tips (**D**). For a more detailed description, see Text S1. Scale bars, 10 μm.
