# Supplementary material for: Dissecting the Nanoscale Distributions and Functions of Microtubule-End-Binding Proteins EB1 and ch-TOG in Interphase HeLa Cells
Source: PLoS One. 2012 Dec 12;7(12):e51442. doi: 10.1371/journal.pone.0051442 (PMC3520847; doi:10.1371/journal.pone.0051442)
Supplement: Figure S5 — Measurement of the relative expression levels of EB1, EB2 and EB3 in HeLa cells. (DOC) [file pone.0051442.s005.doc]

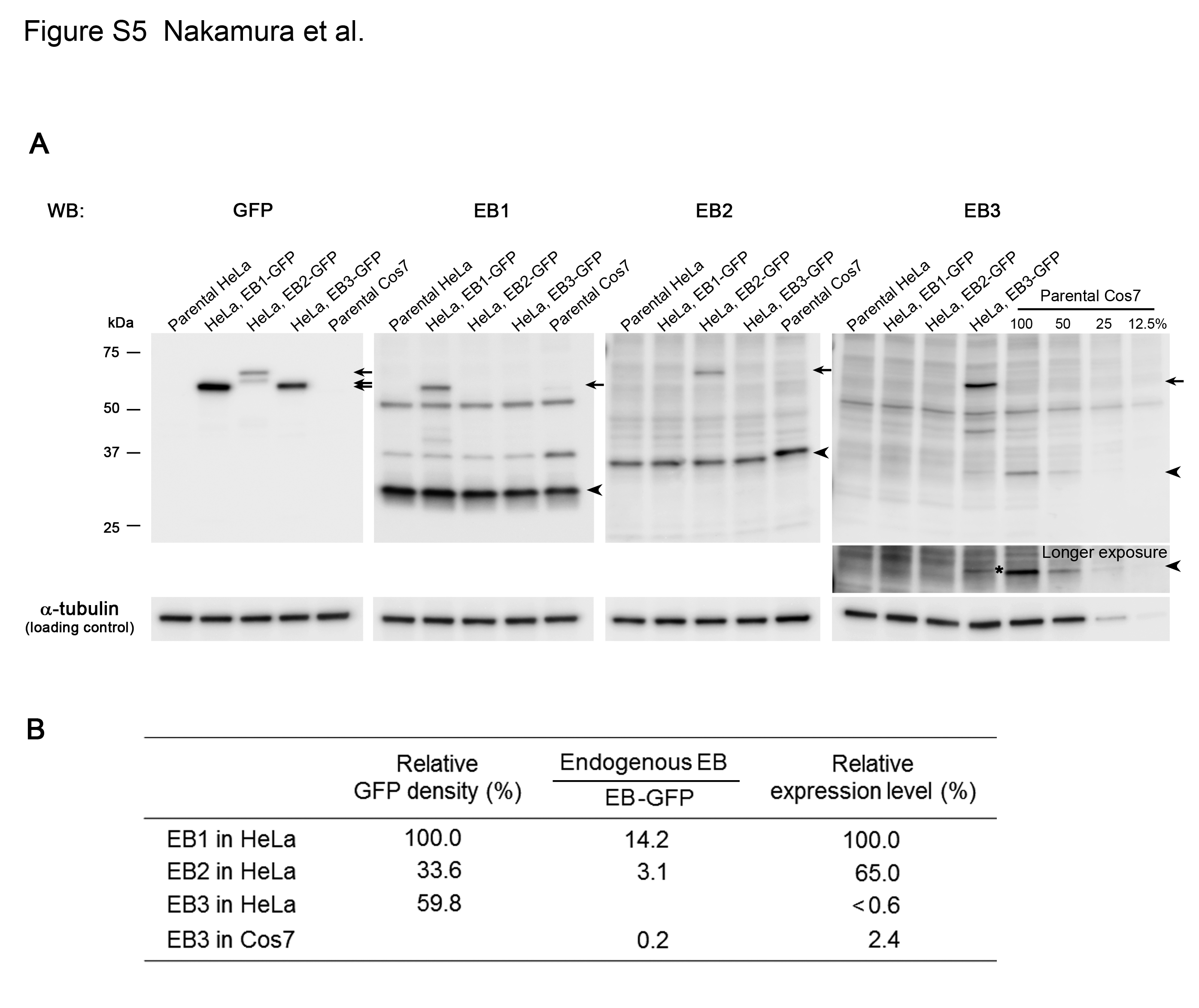


**Figure S5.**

**Measurement of the relative expression levels of EB1, EB2 and EB3 in HeLa cells.** (**A**) HeLa cells were transiently transfected with GFP-fused human EB1, EB2 or EB3 and the cell lysates were lysed and subjected to WB using the antibodies indicated in the figures. A Cos7 cell lysate was used as a positive control for EB3 detection, because endogenous EB3 in HeLa cells is undetectable by WB. For the detection of EB3, a longer exposure image of the same membrane is also shown. The band indicated with an asterisk in the longer exposure image is assumed to be a degraded product of exogenously introduced EB3-GFP, because the corresponding band is not present in the parental HeLa cells. Arrows and arrowheads indicate exogenous and endogenous EB proteins, respectively. (**B**) The band densities of GFP and EB proteins were measured using a dilution series of the cell lysate (data not shown), and the relative expression levels of EB1, EB2 and EB3 were calculated using GFP signals as references. Although endogenous EB3 is undetectable in HeLa cells, its expression level was estimated to be less than 0.6% of endogenous EB1 by using a dilution series of the Cos7 cell lysate. For WB full scans, see also Figure S7D.
