# Supplementary material for: Dissecting the Nanoscale Distributions and Functions of Microtubule-End-Binding Proteins EB1 and ch-TOG in Interphase HeLa Cells
Source: PLoS One. 2012 Dec 12;7(12):e51442. doi: 10.1371/journal.pone.0051442 (PMC3520847; doi:10.1371/journal.pone.0051442)
Supplement: Figure S6 — Full scan western blots 1. (DOC) [file pone.0051442.s006.doc]

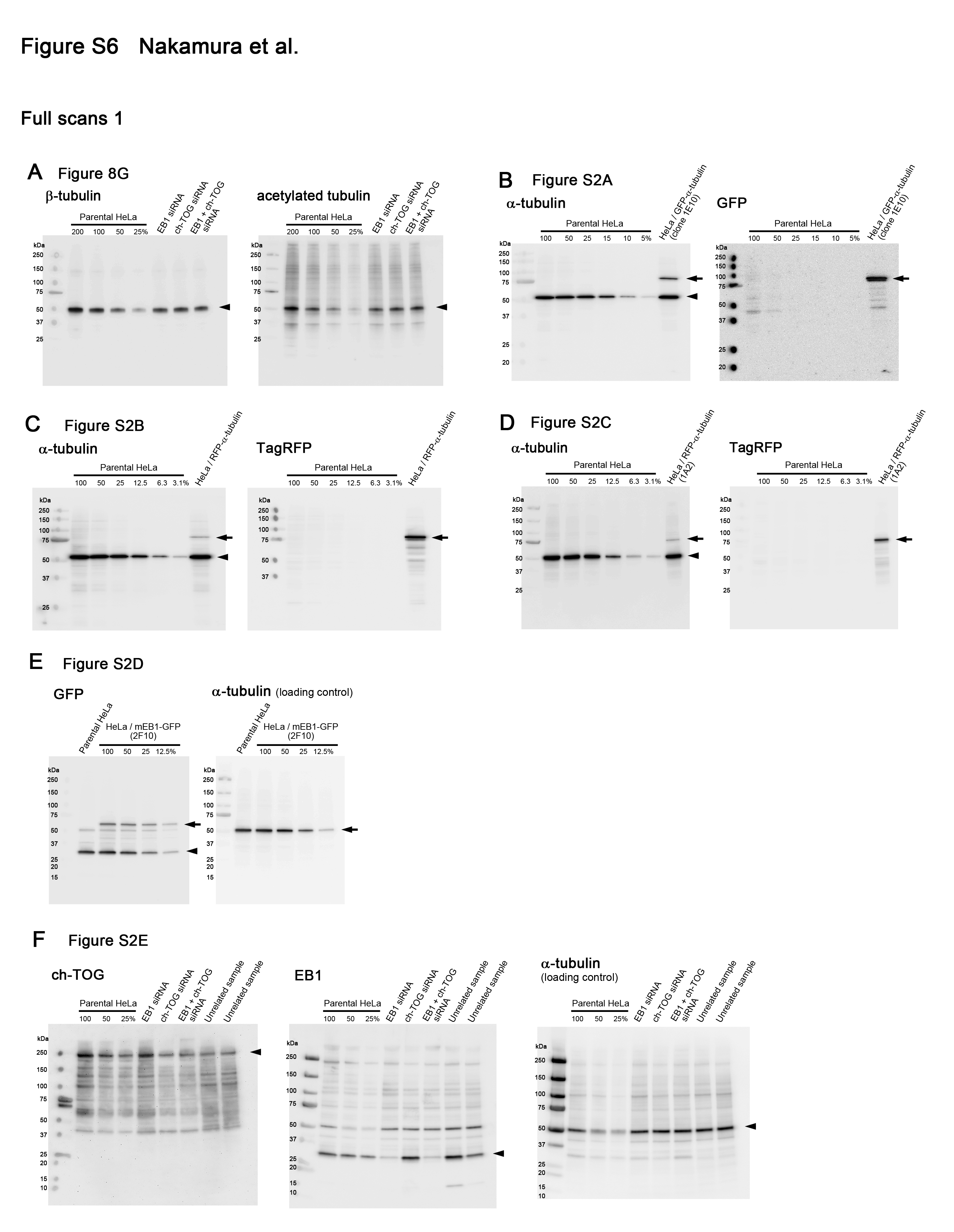
**Figure S6.**

**Full scan western blots 1.** (**A**) Full scan of the blot shown in Figure 8G. (**B**) Full scan of the blot shown in Figure S2A. (**C**) Full scan of the blot shown in Figure S2B. (**D**) Full scan of the blot shown in Figure S2C. (**E**) Full scan of the blot shown in Figure S2D. (**F**) Full scan of the blot shown in Figure S2E. Arrowheads and arrows indicate endogenous and exogenous proteins, respectively.
