# Supplementary material for: Dissecting the Nanoscale Distributions and Functions of Microtubule-End-Binding Proteins EB1 and ch-TOG in Interphase HeLa Cells
Source: PLoS One. 2012 Dec 12;7(12):e51442. doi: 10.1371/journal.pone.0051442 (PMC3520847; doi:10.1371/journal.pone.0051442)
Supplement: Figure S7 — Full scan western blots 2. (DOC) [file pone.0051442.s007.doc]

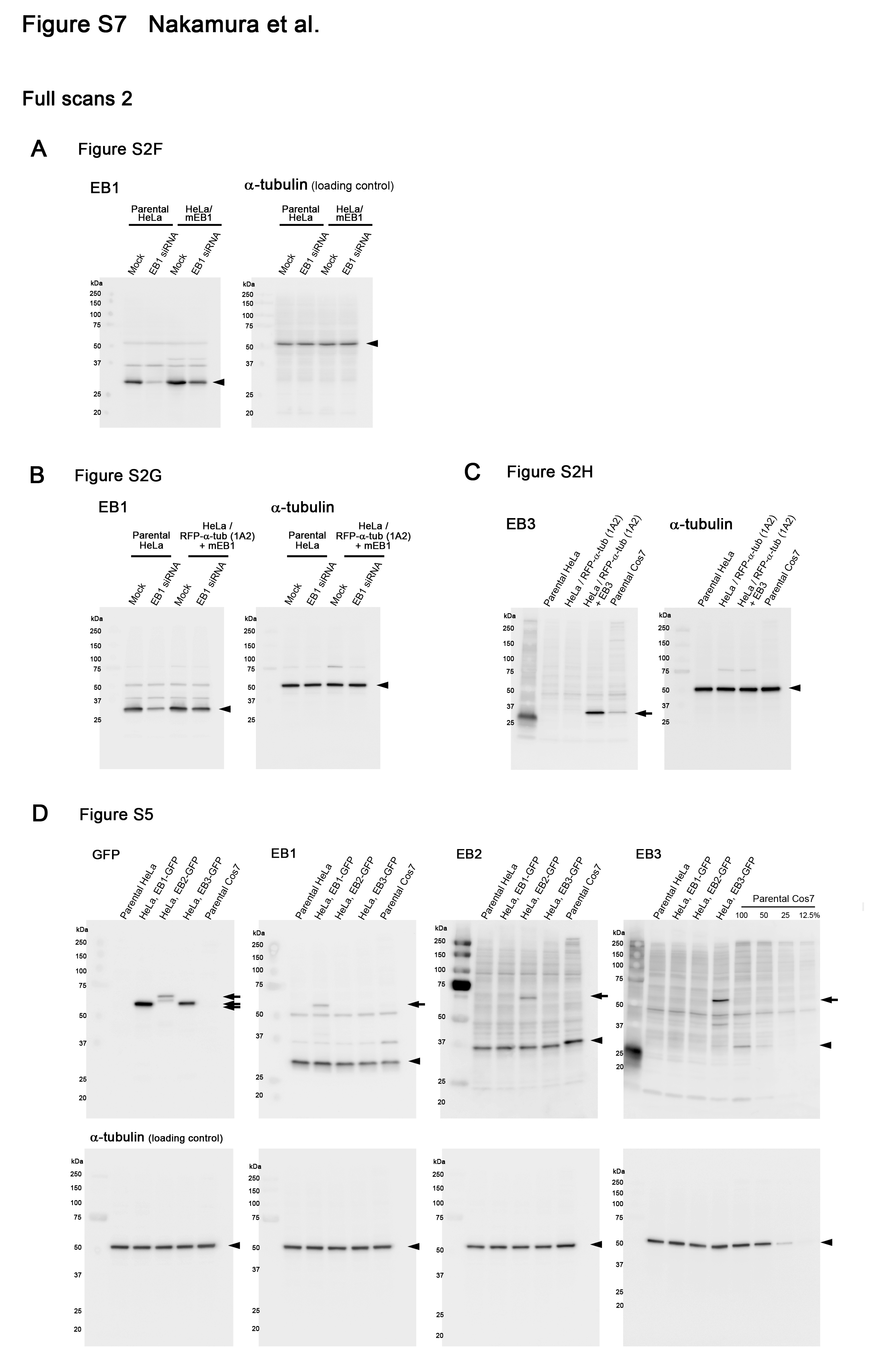


**Figure S7.**

**Full scan western blots 2.** (**A**) Full scan of the blot shown in Figure S2F. (**B**) Full scan of the blot shown in Figure S2G. (**C**) Full scan of the blot shown in Figure S2H. (**D**) Full scan of the blot shown in Figure S5. Arrowheads and arrows indicate endogenous and exogenous proteins, respectively.
