# Supplementary material for: Dissecting the Nanoscale Distributions and Functions of Microtubule-End-Binding Proteins EB1 and ch-TOG in Interphase HeLa Cells
Source: PLoS One. 2012 Dec 12;7(12):e51442. doi: 10.1371/journal.pone.0051442 (PMC3520847; doi:10.1371/journal.pone.0051442)
Supplement: Text S1 — Supplemental Results and Discussion. (DOC) [file pone.0051442.s013.doc]

**Supplemental Results and Discussion**

**Different microtubule organisations in cells seeded on collagen-coated vs. uncoated glass coverslips**

We previously reported that in HeLa cells seeded on uncoated coverslips, many microtubule plus ends terminate in the narrow region (~2 μm) near the cell periphery, where CLASP1 and CLASP2, which are potent microtubule-anchoring factors that associate with EB1 and microtubules, accumulate in a LL5-dependent manner [1,2]. LL5 proteins are a family of PH-domain-containing coiled-coil proteins that includes LL5α, LL5β and LL5γ (also known as pleckstrin homology-like domain, family B, member 1 (PHLDB1)), PHLDB2 and PHLDB3, while PHLDB3 is a non-conserved short protein carrying only a PH domain. LL5α and LL5β, which are recruited to the cell cortex upon the binding of integrins to the ECM, attach to microtubules via the CLASP-EB1 complex [3].

When HeLa cells were seeded onto collagen-coated coverslips, the cells quickly spread, CLASPs were observed to be distributed more diffusely near the cell periphery, and the microtubule density was reduced (Figure S1). Because the peripheral microtubules on uncoated coverslips were too dense to enable the visualisation and analysis of individual filaments separately at high resolution, we cultured cells on collagen-coated glass in this study.

**Precise visualisation of microtubule ends**

We were aware that immunostaining of microtubules using anti-tubulin antibody was weakened at the very ends of microtubules where EB1 comets can be labelled (Figure S3), probably owing to antigen masking by condensed accumulation of microtubule-binding proteins. Therefore, immunostaining is not suitable for determining the precise position of the microtubule ends. Instead, we used HeLa cells expressing GFP-α-tubulin (clone 1E10). The expression level of exogenous GFP-fused α-tubulin in these cells is ~12% of that of endogenous tubulin (Figure S2A), and thus on average approximately 1.5 GFP molecules are estimated to be incorporated per turn of the tube (which consists of 13 dimers). The GFP-α-tubulin signals enabled us to visualise entire microtubules without signal decay at their ends (Figure S3E, F).

**Comparison of the distributions of GFP-fused ch-TOG constructs and endogenous ch-TOG**

In previous studies, NH_2_-terminal GFP fusion proteins (GFP-XMAP215 and GFP-ch-TOG) have been used to visualise XMAP215 and ch-TOG in cells [4,5]. In these studies, XMAP215 and ch-TOG were distributed along the microtubule lattice and their tracking on polymerising microtubule tips was undetectable. However, when GFP-ch-TOG was co-expressed with SLAIN2, it tracked on growing microtubule ends [6]. Also, using our GFP-ch-TOG construct (Figure S4A-C), GFP-ch-TOG was prone to distribute along the entire length of microtubules when its expression level was high in HeLa cells, although at lower expression levels it was detectable at the very ends of microtubules by TIRF microscopy (Figure S4B, C). In contrast, the COOH-terminal GFP-fusion protein, ch-TOG-GFP, only very weakly binds to the microtubule lattice even upon high-level overexpression (Figure S4E, F), similar to endogenous ch-TOG. When comparing the distributions of mEB1-RFP and GFP-fused ch-TOG, ch-TOG-GFP was localised to the majority of tips of EB1 comets (Figure 4A), whereas GFP-ch-TOG was found only at a subset of EB1 comets (Figure S4D). Live imaging revealed that GFP-ch-TOG tracks shortening microtubule ends, but is rarely found at growing microtubule ends (data not shown). It is assumed that the GFP-ch-TOG fusion protein is dysfunctional. Brouhard et al. successfully visualised the tracking of XMAP215 on growing and shortening microtubule ends in an *in vitro* study using a COOH-terminal GFP fusion of XMAP215 [7]. Also, in the case of the *Drosophila* XMAP215 homologue, Mini spindles (Msps), the COOH-terminal GFP fusion protein tracked growing and shortening microtubule ends in cells [8,9]. Taken together, these data suggest that a COOH-terminal GFP tag does not disrupt the innate function of this protein family, and we therefore used the ch-TOG-GFP construct in this study.

We noted a difference in the distribution patterns of ch-TOG in the cytoplasm of living and fixed cells. In living cells, the ch-TOG-GFP clusters were observed only at the microtubule ends and not in the cytoplasm, while extensive diffuse signals were observed in the cytoplasm (Figure 2; Figure S6E, F). This is in contrast to the immunostaining of endogenous ch-TOG, in which ch-TOG spots are observed throughout the cytoplasm (Figure 1A-C). However, when the ch-TOG-GFP-expressing cells were fixed, granular patterns were observed in the cytoplasm (Figure 6D). Presumably the cytoplasmic ch-TOG pool that is undergoing fast diffusive motion without attaching to the microtubules cannot be imaged as separated signals but creates diffuse background signals. Alternatively, diffuse ch-TOG forms clusters during the fixation processes. In addition, the specimens prepared by the immunostaining procedure used in this study contain ~30% non-specific background noise (Figure 3). However, the localisation of ch-TOG to the microtubule tips was observed consistently both in living and fixed cells. Thus, we can obtain a precise localisation of ch-TOG at the microtubule tips, particularly by application of the averaging procedure.

**Supplemental References**

1. Mimori-Kiyosue Y, Grigoriev I, Lansbergen G, Sasaki H, Matsui C, et al. (2005) CLASP1 and CLASP2 bind to EB1 and regulate microtubule plus-end dynamics at the cell cortex. J Cell Biol 168: 141-153.

2. Lansbergen G, Grigoriev I, Mimori-Kiyosue Y, Ohtsuka T, Higa S, et al. (2006) CLASPs attach microtubule plus ends to the cell cortex through a complex with LL5 beta. Developmental Cell 11: 21-32.

3. Hotta A, Kawakatsu T, Nakatani T, Sato T, Matsui C, et al. (2010) Laminin-based cell adhesion anchors microtubule plus ends to the epithelial cell basal cortex through LL5alpha/beta. J Cell Biol 189: 901-917.

4. Popov AV, Pozniakovsky A, Arnal I, Antony C, Ashford AJ, et al. (2001) XMAP215 regulates microtubule dynamics through two distinct domains. EMBO J 20: 397-410.

5. Moore AT, Rankin KE, von Dassow G, Peris L, Wagenbach M, et al. (2005) MCAK associates with the tips of polymerizing microtubules. J Cell Biol 169: 391-397.

6. van der Vaart B, Manatschal C, Grigoriev I, Olieric V, Gouveia SM, et al. (2011) SLAIN2 links microtubule plus end-tracking proteins and controls microtubule growth in interphase. J Cell Biol 193: 1083-1099.

7. Brouhard GJ, Stear JH, Noetzel TL, Al-Bassam J, Kinoshita K, et al. (2008) XMAP215 is a processive microtubule polymerase. Cell 132: 79-88.

8. Li W, Miki T, Watanabe T, Kakeno M, Sugiyama I, et al. (2011) EB1 promotes microtubule dynamics by recruiting Sentin in Drosophila cells. J Cell Biol 193: 973-983.

9. Currie JD, Stewman S, Schimizzi G, Slep KC, Ma A, et al. (2011) The microtubule lattice and plus-end association of Drosophila Mini spindles is spatially regulated to fine-tune microtubule dynamics. Molecular Biology of the Cell 22: 4343-4361.
